# Systemic Risk in a Multilayer Financial Network: A Criticality-Based Perspective

**DOI:** 10.3390/e28101148

**Published:** 2026-10-19

**Authors:** Ning Xi, Yamin Dou, Yazheng Dang

**Affiliations:** Department of Systems Science, Business School, University of Shanghai for Science and Technology, Shanghai 200093, China; nxi@usst.edu.cn (N.X.); dym1182022@163.com (Y.D.)

**Keywords:** system-wide collapse, criticality, multilayer financial network, default contagion, systemic risk, interfirm trade credit

## Abstract

Banks and firms are connected through multiple credit relationships that can transmit financial shocks across sectors, amplify default contagion, and ultimately lead to system-wide collapse. Understanding systemic risk therefore requires not only examining default contagion mechanisms but also characterizing system-wide collapse. To this end, we construct a three-layer financial network model and examine its response to exogenous shocks. Under the baseline configuration, numerical simulations reveal pronounced bimodal distributions of bankruptcy fractions, indicating that the sharp increase in average bankruptcy fractions mainly reflects a shift in the relative frequencies of limited-contagion and system-wide collapse states. Susceptibility and finite-size analyses provide further evidence of phase-transition-like behavior. Building on these findings, we characterize systemic risk using complementary measures of collapse likelihood, shock tolerance, and financial damage, measured by system-wide collapse probability, collapse thresholds, and layer-specific loss ratios, respectively. Matched counterfactual experiments show that two-layer configurations without endogenous interfirm credit propagation have higher system-wide collapse thresholds than the three-layer model under both recovery-matching conventions, although the increase is much larger under recovery-rate matching than under recovery-amount matching. Parameter experiments further show how balance-sheet composition, interfirm link formation, and credit exposure are associated with shock tolerance.

## 1. Introduction

The 2008 global financial crisis precipitated severe financial turmoil and a deep global recession, shifting the focus of academic and institutional discourse toward understanding and mitigating systemic risk [1]. In the years that followed, however, the global financial system was repeatedly tested by major shocks, underscoring the persistence of these vulnerabilities. The COVID-19 pandemic, for example, inflicted profound and widespread damage on global finance [2]: lockdown measures and supply chain disruptions produced acute economic stagnation and dramatic market volatility, renewing concerns about systemic risk. More recently, the failure of Silicon Valley Bank and the emergency rescue of Credit Suisse exposed critical structural weaknesses within the financial system, triggering contagion effects that reverberated through depositors, borrowers, cryptocurrency markets, and interconnected banks [3,4,5]. Although these banking stress events did not escalate into a full-blown global crisis comparable to 2008, they caused substantial disruptions to financial markets and the broader economy. These episodes highlight the importance of understanding how interconnected financial relationships transmit and amplify distress, potentially leading to system-wide collapse.

Early research on financial networks and systemic risk focused primarily on interbank networks, in which banks are connected through direct credit exposures. Allen and Gale were among the first to introduce network structure into the study of systemic risk [6]. By analyzing a simple four-bank network, they found that under their model assumptions, a complete network is more resilient than an incomplete one. Building on similar simplified frameworks, subsequent studies have confirmed that network structure significantly affects systemic risk in banking systems [7,8]. The development of complex network science has provided a broad set of analytical tools for systemic risk research, enabling theoretical modeling, empirical characterization, and quantitative risk assessment of financial networks. Theoretically, researchers have developed multi-agent models based on network theory, comparing systemic risk across interbank networks with different statistical properties [9,10,11]. Empirically, analyses using real bank data have shown that interbank networks often display small-world or scale-free topological features [12,13,14,15]. Additionally, researchers have conducted quantitative assessments of systemic risk in specific national interbank networks. For example, Upper and Worms estimated that the failure of a single bank in Germany could lead to a breakdown affecting up to 15% of the system’s assets [16], while Degryse and Nguyen found that the Belgian banking system’s evolution from a complete structure to a multiple-money-center network structure helped reduce contagion risk [17].

These studies have primarily examined single-layer interbank networks, in which only one type of financial relationship is modeled at a time. However, in reality, banks are connected by multiple types of financial relationships, each of which can be represented as a distinct network layer. For instance, Langfield et al. distinguished interbank exposure and interbank funding networks as separate layers within a multilayer interbank network, using financial instruments extracted from UK regulatory data [18]. Risk propagation in multilayer networks can exhibit non-additive effects, whereby the combined contagion effects across layers may substantially exceed the sum of individual-layer effects [19], a finding supported by empirical evidence [20]. Moreover, Bargigli et al. demonstrated that aggregating different network layers can lead to biased estimates of systemic risk [21]. These findings indicate that multilayer network models can better capture distinct channels of risk transmission and reduce biases arising from the aggregation of different network layers. A comprehensive review of financial networks, encompassing both single-layer and multilayer frameworks, is provided by Bardoscia et al. [22].

Although multilayer frameworks have improved the modeling of interbank relationships, the role of firms in systemic risk remains comparatively underexplored. In modern economies, firms are tightly connected to banks through credit relationships, so shocks affecting firms can be transmitted to banks and further amplified through interbank linkages. Using Brazilian data, empirical studies have constructed multilayer networks that include both bank–firm and interbank relationships, showing that firm distress can propagate through the banking network and highlighting the importance of incorporating firms into systemic risk estimation [23,24]. Lux proposed a random bipartite model of bank–firm credit relationships that reproduces key statistical regularities observed in loan data across multiple countries. Simulation results indicate that the bank–firm lending channel can play a more important role than the interbank lending channel in default propagation [25]. Building on this model, Gao introduced bank asset portfolios and examined how systemic risk evolves under different shock types, including firm defaults, asset devaluation, and bank failures [26]. Nevertheless, direct interfirm credit relationships remain insufficiently incorporated in most bank–firm network studies. In reality, firms are embedded in large-scale production networks, acting simultaneously as customers and suppliers. Since suppliers frequently extend trade credit to their customers, production linkages are commonly accompanied by interfirm credit exposures, creating an additional channel through which credit shocks can propagate. A growing body of research has documented important statistical and dynamic features of production and interfirm networks [27,28,29,30,31,32,33,34,35,36,37]. Notably, an empirical study based on Uruguayan data shows that neglecting interfirm credit exposures can lead to a substantial underestimation of systemic risk [38].

In this context, Li and Sui constructed an endogenous financial network model incorporating interbank, bank–firm, and interfirm relationships. By introducing different partner selection mechanisms, they examined how network evolution influences default contagion [39]. Their work represents an important step toward incorporating interfirm relationships into multilayer models of default contagion. Subsequent studies have further extended multilayer bank–firm modeling frameworks. For example, Jin et al. developed a dynamic multilayer financial network model incorporating interbank lending, bank–firm credit relationships, and cross-shareholding among firms, and examined how network structure and attack strategies influence systemic stability [40]. Similarly, Lei et al. constructed a multilayer bank–firm network integrating interbank lending, bank–firm credit, interfirm trade credit, guarantee relationships, and cross-holdings and analyzed how different types of shocks propagate through these interconnected channels [41]. Taken together, these studies have examined how network structure, credit relationships, and shock mechanisms affect default contagion in multilayer financial networks. However, the statistical characteristics of system-wide collapse remain less well understood in these models. A sharp increase in average bankruptcy fractions alone cannot distinguish a gradual shift in the distribution toward higher values from a change in the relative frequencies of distinct states, nor does it establish how system-wide collapse behavior depends on system size. These questions motivate a criticality-based analysis of system-wide collapse.

To address these questions, we adopt a criticality-based perspective to analyze systemic risk in multilayer financial networks. Building on the Lux model, we construct a three-layer financial network comprising interbank lending, bank–firm lending, and interfirm trade credit within a unified balance-sheet framework. Treating external shock intensity as a control parameter, we examine bankruptcy-fraction distributions, susceptibility, and finite-size behavior to characterize system-wide collapse. We then use three complementary measures to characterize systemic risk: system-wide collapse probability to quantify collapse likelihood at a given shock intensity; the system-wide collapse threshold to characterize shock tolerance, supplemented by sector-specific collapse thresholds to distinguish bank and firm responses; and layer-specific loss ratios to capture the severity of financial damage.

Within this framework, we investigate the contribution of endogenous interfirm credit propagation and the relationships between selected balance-sheet and interfirm-layer parameters and shock tolerance. Matched counterfactual experiments show that removing endogenous interfirm credit propagation raises the system-wide collapse threshold under both recovery-matching conventions, although the magnitude of the increase depends on how recovery resources are matched. We further examine how bank capitalization, firm cash holdings, interfirm link formation, and credit exposure are associated with shock tolerance and sectoral responses.

The remainder of this paper is organized as follows. Section 2 presents the multilayer financial network model and default contagion mechanisms. Section 3 examines system-wide collapse behavior, system-wide collapse probabilities, system-wide and sector-specific collapse thresholds, and layer-specific loss ratios, followed by finite-size analysis, matched counterfactual comparisons of endogenous interfirm credit propagation, balance-sheet and interfirm-layer parameter experiments, and robustness checks. Section 4 summarizes the main findings and discusses the study’s limitations.

## 2. Model

We model a financial system consisting of banks and firms that are connected through three types of credit relationships, each represented as a distinct network layer. Banks provide loans to firms, forming the bank–firm network; banks engage in interbank lending, forming the interbank network; and firms transact with other firms through trade credit, forming the interfirm network. These financial activities are captured in the balance sheets of banks and firms. By leveraging the accounting identity embedded in the balance sheets, the three networks are structurally coupled, jointly forming a complex multilayer financial network.

### 2.1. Bank–Firm Network

Empirical studies have identified several stylized characteristics of bank–firm networks. Axtell’s analysis of U.S. firms shows that firm size follows a power-law distribution with an exponent close to one [42]. Similarly, Bremus et al. analyzed cross-country data and found that the right tail of the bank size distribution exhibits a power-law pattern [43]. De Masi and Gallegati, based on their study of the Italian market, drew two key conclusions regarding the bank–firm network. First, banks have a much higher average degree than firms. Second, connectivity is heterogeneous and exhibits clear size-related patterns on both sides: bank degree is strongly associated with bank size, while large firms tend to be financed by multiple banks [44].

Motivated by these empirical findings, we adopt the network-generation algorithm proposed by Lux to construct the bank–firm layer [25]. The statistical properties of the networks generated under our baseline parameter settings are evaluated separately in Section 3.1.1. Let $N_{b}$ denote the number of banks, $N_{f}$ the number of firms, and $\lambda_{f}$ the average degree of firms in the bank–firm network. The average degree of banks is then given by $\lambda_{b}=\lambda_{f}N_{f}/N_{b}$. For the banking sector, we assume that the total assets $A_{i}^{b}$ ($i=1,2,\ldots ,N_{b}$) of bank *i* follow a truncated Pareto distribution with the probability density function:

(1) $$f\left(A_{i}^{b}\right)=\frac{\alpha {\left(A_{b}^{LB}\right)}^{\alpha }{\left(A_{i}^{b}\right)}^{-\alpha -1}}{1-{\left(A_{b}^{LB}/A_{b}^{UB}\right)}^{\alpha }},$$

where $A_{b}^{LB}$ and $A_{b}^{UB}$ represent the lower and upper bounds of bank total assets, respectively, and α is the Pareto exponent. The total assets of each bank are generated by sampling from this distribution. In line with the empirical findings of De Masi and Gallegati [44], we assume that the expected degree ${\tilde{\lambda }}_{i}^{b}$ of bank *i* is proportional to its total assets $A_{i}^{b}$, such that ${\tilde{\lambda }}_{i}^{b}=\lambda_{b}A_{i}^{b}/{\bar{A}}_{b}$, where ${\bar{A}}_{b}$ denotes the sample mean of bank total assets. Furthermore, for simplicity, we assume that each bank allocates a fixed proportion $\theta_{bf}$ of its total assets to total loans to firms, as follows:

(2) $$L_{i}^{bf}=\theta_{bf}A_{i}^{b},$$

where $\theta_{bf}$ is identical across banks. Since firm assets often serve as an important basis for bank lending, firms’ total bank loans tend to increase with their total assets. Consequently, the distribution of total bank loans across firms is expected to exhibit a similar shape to the distribution of firms’ total assets, which follows a power-law distribution. Accordingly, for the firm sector, we assume that the expected total bank loans ${\tilde{L}}_{j}^{fb}$ ($j=1,2,\ldots ,N_{f}$) of firm *j* follow a distribution analogous to Equation (1), with the same Pareto exponent α, but with modified bounds. Specifically, the lower and upper bounds are defined as $L_{fb}^{LB}=\theta_{bf}A_{b}^{LB}N_{b}/N_{f}$ and $L_{fb}^{UB}=\theta_{bf}A_{b}^{UB}N_{b}/N_{f}$, respectively. These bounds specify the range of expected total bank loans for each firm. The expected total bank loans for each firm are generated by sampling from this distribution. We further assume that the expected degree ${\tilde{\lambda }}_{j}^{f}$ of firm *j* is proportional to its expected total bank loans: ${\tilde{\lambda }}_{j}^{f}=\lambda_{f}{\tilde{L}}_{j}^{fb}/{\bar{\tilde{L}}}^{fb}$, where ${\bar{\tilde{L}}}^{fb}$ denotes the sample mean of firms’ expected total bank loans.

By construction, the sums of expected degrees on the bank and firm sides are equal: $\sum_{i}{\tilde{\lambda }}_{i}^{b}=\lambda_{b}N_{b}=\lambda_{f}N_{f}=\sum_{j}{\tilde{\lambda }}_{j}^{f}$. We therefore set the target number of links to $\lambda_{f}N_{f}$. The network is then constructed using weighted random sampling. Specifically, at each step, a bank and a firm are selected independently, with probabilities proportional to their respective expected degrees. A link is established only if the selected pair is not already connected. This iterative process continues until the target number of links is reached. To further ensure that each entity has at least one link, each unconnected bank is connected to a firm selected with probability proportional to the firm’s expected degree. Similarly, each unconnected firm is connected to a bank selected with probability proportional to the bank’s expected degree. In the resulting network, all links are directed from banks to firms, indicating loan provision from banks to firms. This structure is represented by a directed adjacency matrix $M^{bf}=\left[m_{ij}^{bf}\right]$ of dimension $N_{b}\times N_{f}$, where $m_{ij}^{bf}=1$ if a directed link from bank *i* to firm *j* exists, and 0 otherwise. We assume that each bank distributes its total loans to firms equally across all its connected firms. Accordingly, the loan from bank *i* to firm *j* is given by

(3) $$L_{ij}^{bf}=\frac{L_{i}^{bf}m_{ij}^{bf}}{\lambda_{i}^{b}},$$

where $\lambda_{i}^{b}=\sum_{j=1}^{N_{f}}m_{ij}^{bf}$ represents the degree of bank *i*. It follows that the total bank loans of firm *j* are calculated as

(4) $$L_{j}^{fb}=\sum_{i=1}^{N_{b}}L_{ij}^{bf}.$$

The resulting total bank loans of firms are determined by the generated network and the equal-allocation rule and may differ from their sampled expected values.

### 2.2. Interbank Network

Empirical studies have examined the degree distributions and mixing patterns of interbank networks [12,13]. Motivated by these studies, Montagna and Lux proposed a Monte Carlo framework for generating interbank networks with asset-dependent link formation [45]. This algorithm has been widely applied in studies on systemic risk in the banking sector [25,26]. We adopt this framework to construct the interbank layer. Its realized degree distributions and mixing patterns under our baseline settings are examined in Section 3.1.1. The probability of a directed link from bank *i* to bank *j*—representing an interbank loan extended by bank *i* to bank *j*—is given by

(5) $$p_{ij}^{bb}=d_{b}{\left(\frac{A_{i}^{b}}{A_{b}^{\max}}\right)}^{\alpha_{1}}{\left(\frac{A_{j}^{b}}{A_{b}^{\max}}\right)}^{\alpha_{2}},$$

where $\alpha_{1}$, $\alpha_{2}$, and $d_{b}$ are parameters; $A_{i}^{b}$ and $A_{j}^{b}$ denote the total assets of bank *i* and bank *j*, respectively; and $A_{b}^{\max}$ represents the maximum total assets among all banks. Subsequently, the directed adjacency matrix $M^{bb}$ of the interbank network is constructed, where diagonal entries are set to zero, and off-diagonal entries are determined by

(6) $$m_{ij}^{bb}=\left\{\begin{array}{cc} 1, & \mathrm{with}\;\mathrm{probability}\;p_{ij}^{bb}\\ 0, & \mathrm{with}\;\mathrm{probability}\;1-p_{ij}^{bb} \end{array}\right..$$

To ensure that each bank has at least one outgoing link, we assign an outgoing link from each bank with zero out-degree to another bank chosen with probability proportional to its total assets. The adjacency matrix $M^{bb}$ is updated accordingly.

After establishing the network topology, we define the weights of the credit relationships. For a given bank, the total loans it extends to other banks are referred to as interbank lending, while the total loans it receives from other banks are referred to as interbank borrowing. To simplify the model, we assume that each bank allocates a fixed proportion $\theta_{bb}$ of its total assets to interbank lending:

(7) $$L_{i}^{IBL}=\theta_{bb}A_{i}^{b},$$

where $\theta_{bb}$ is identical across banks. Furthermore, we assume that the interbank lending of bank *i* is allocated among borrowing banks in proportion to their total assets. Thus, the interbank loan from bank *i* to bank *j* is given by

(8) $$L_{ij}^{bb}=L_{i}^{IBL}\frac{A_{j}^{b}m_{ij}^{bb}}{\sum_{h=1}^{N_{b}}A_{h}^{b}m_{ih}^{bb}}.$$

Accordingly, the interbank borrowing of bank *i* can be expressed as

(9) $$L_{i}^{IBB}=\sum_{k=1}^{N_{b}}L_{ki}^{bb}.$$

### 2.3. Interfirm Network

Empirical studies have investigated heterogeneous connectivity and size-related linking patterns in interfirm networks [46,47,48]. Motivated by these findings, we adopt the asset-dependent network-generation framework of Montagna and Lux to construct the interfirm layer [45]. The properties of the resulting network are evaluated in Section 3.1.1. The construction process begins by determining the total assets of each firm. For simplicity, we assume that firms’ total bank loans are proportional to their total assets. Given firm *j*’s total bank loans $L_{j}^{fb}$ in Equation (4), its total assets can then be inferred as

(10) $$A_{j}^{f}=\frac{L_{j}^{fb}}{\mu_{fb}},$$

where $\mu_{fb}$ represents the share of a firm’s total bank loans in its total assets and is assumed to be identical across firms. Next, the probability of a directed link from firm *i* to firm *j* is defined as

(11) $$p_{ij}^{ff}=d_{f}{\left(\frac{A_{i}^{f}}{A_{f}^{\max}}\right)}^{\alpha_{1}}{\left(\frac{A_{j}^{f}}{A_{f}^{\max}}\right)}^{\alpha_{2}},$$

where $\alpha_{1}$, $\alpha_{2}$, and $d_{f}$ are parameters; $A_{i}^{f}$ and $A_{j}^{f}$ denote the total assets of firm *i* and firm *j*, respectively; and $A_{f}^{\max}$ represents the maximum total assets among all firms. Here, a directed link from firm *i* to firm *j* indicates that firm *i* is the supplier and firm *j* is the customer, with firm *i* extending trade credit to firm *j*. For simplicity, $\alpha_{1}$ and $\alpha_{2}$ are set equal to those in Equation (5). Based on this probability, the directed adjacency matrix $M^{ff}$ of the interfirm network is constructed with diagonal entries equal to 0 and off-diagonal entries determined by

(12) $$m_{ij}^{ff}=\left\{\begin{array}{cc} 1, & \mathrm{with}\;\mathrm{probability}\;p_{ij}^{ff}\\ 0, & \mathrm{with}\;\mathrm{probability}\;1-p_{ij}^{ff} \end{array}\right..$$

To ensure that each firm has at least one incoming link, any firm with zero in-degree receives a directed link from another firm selected with probability proportional to its total assets. The adjacency matrix $M^{ff}$ is updated accordingly.

In real-world economies, transactions between firms are often conducted through trade credit. Under such arrangements, suppliers deliver goods or services first, and customers settle payments at a later, pre-agreed date. This practice essentially constitutes a form of short-term financing, recorded as accounts receivable on suppliers’ balance sheets and as accounts payable on customers’ balance sheets. For simplicity, we assume that firm *j*, acting as a customer, has accounts payable equal to a fixed proportion $\mu_{ff}$ of its total assets. Accordingly, firm *j*’s accounts payable are given by

(13) $$L_{j}^{AP}=\mu_{ff}A_{j}^{f},$$

where $\mu_{ff}$ is identical across firms. Moreover, we assume that firm *j* allocates its accounts payable among its suppliers in proportion to their total assets. Therefore, the trade credit extended by firm *i* to firm *j* is determined as follows:

(14) $$L_{ij}^{ff}=L_{j}^{AP}\frac{A_{i}^{f}m_{ij}^{ff}}{\sum_{h=1}^{N_{f}}A_{h}^{f}m_{hj}^{ff}}.$$

Consequently, firm *i*’s accounts receivable are obtained as the sum of all such trade credits extended to its customers:

(15) $$L_{i}^{AR}=\sum_{j=1}^{N_{f}}L_{ij}^{ff}.$$

### 2.4. Balance Sheets of Banks and Firms

The balance sheet presents the financial position of banks and firms through a set of financial variables and is constructed in accordance with the fundamental accounting identity that total assets equal total liabilities. Its structure comprises distinct categories for assets and liabilities. Based on the model setup, we simplify the conventional balance sheet while ensuring that it still accurately captures the key financial characteristics of banks and firms.

The balance sheet of bank *i* is shown in Table 1. On the asset side, the total assets $A_{i}^{b}$ of bank *i* comprise total loans to firms $L_{i}^{bf}$, interbank lending $L_{i}^{\mathrm{IBL}}$, and cash $C_{i}^{b}$, as expressed by

(16) $$A_{i}^{b}=L_{i}^{bf}+L_{i}^{\mathrm{IBL}}+C_{i}^{b}.$$

On the liability side, the total liabilities $L_{i}^{b}$ of bank *i* consist of equity $E_{i}^{b}$, interbank borrowing $L_{i}^{\mathrm{IBB}}$, and deposits $D_{i}^{b}$, as given by

(17) $$L_{i}^{b}=E_{i}^{b}+L_{i}^{\mathrm{IBB}}+D_{i}^{b}.$$

As required by the accounting identity, the total assets and total liabilities of bank *i* must be equal, yielding

(18) $$A_{i}^{b}=L_{i}^{b}.$$

The variables are specified as follows. The total assets $A_{i}^{b}$ are obtained by sampling from the truncated Pareto distribution defined in Equation (1). The total loans to firms $L_{i}^{bf}$ are computed using Equation (2). The interbank lending $L_{i}^{\mathrm{IBL}}$ is derived from Equation (7). Cash $C_{i}^{b}$ is determined from Equation (16). According to Equation (18), the total liabilities $L_{i}^{b}$ are equal to the total assets $A_{i}^{b}$. For simplicity, we assume that the share of equity in total assets, denoted by $\omega_{b}$, is identical across banks. The equity is therefore expressed as

(19) $$E_{i}^{b}=\omega_{b}A_{i}^{b}.$$

The interbank borrowing $L_{i}^{\mathrm{IBB}}$ is obtained from Equation (9). The deposits $D_{i}^{b}$ are determined from Equation (17). Therefore, once the three network layers are established, the balance sheets of all banks can be generated to represent their financial positions.

The balance sheet of firm *j* is shown in Table 2. On the asset side, the total assets $A_{j}^{f}$ of firm *j* comprise accounts receivable $L_{j}^{AR}$, fixed assets $K_{j}^{f}$, and cash $C_{j}^{f}$, as defined by

(20) $$A_{j}^{f}=L_{j}^{AR}+K_{j}^{f}+C_{j}^{f}.$$

On the liability side, the total liabilities $L_{j}^{f}$ of firm *j* consist of equity $E_{j}^{f}$, accounts payable $L_{j}^{AP}$, and total bank loans $L_{j}^{fb}$, as formulated in

(21) $$L_{j}^{f}=E_{j}^{f}+L_{j}^{AP}+L_{j}^{fb}.$$

In accordance with the accounting identity, the total assets and total liabilities of firm *j* must be equal, leading to

(22) $$A_{j}^{f}=L_{j}^{f}.$$

The variables are specified as follows. The total assets $A_{j}^{f}$ are obtained from Equation (10). The accounts receivable $L_{j}^{AR}$ are given by Equation (15). Let $\mu_{c}$ denote the share of cash in total assets. Cash is then calculated as

(23) $$C_{j}^{f}=\mu_{c}A_{j}^{f},$$

where $\mu_{c}$ is identical across firms. The fixed assets $K_{j}^{f}$ can be determined from Equation (20). Based on Equation (22), the total liabilities $L_{j}^{f}$ are equal to the total assets $A_{j}^{f}$. The accounts payable $L_{j}^{AP}$ are specified by Equation (13). The total bank loans $L_{j}^{fb}$ are computed using Equation (4). The equity $E_{j}^{f}$ is then derived from Equation (21). Therefore, after constructing the three network layers, the balance sheets of all firms can be generated to represent their financial positions. To ensure balance-sheet feasibility, candidate financial configurations with negative bank deposits, firm fixed assets, or firm equity are rejected, and the network and associated balance sheets are regenerated until these quantities are nonnegative. Section 2.1, Section 2.2, Section 2.3 and Section 2.4 describe the initial configuration of the financial system, including the three network layers and the balance sheets of banks and firms. For simplicity, all variables in this part are presented without explicit time indices and refer to their initial values.

### 2.5. Default Contagion Mechanism

We model the contagion dynamics as a discrete-time iterative process. Hereafter, the variables introduced in the preceding sections evolve dynamically as the contagion process unfolds. Within each iteration, events such as liquidations, recoveries, repayments, and defaults occur sequentially, and the affected variables are updated instantaneously. These updated values then serve as inputs for subsequent calculations within the same iteration. Consequently, certain balance-sheet items—such as banks’ cash assets, total loans to firms, and firms’ accounts receivable and accounts payable—may undergo multiple updates within a single iteration as different events occur. To maintain conciseness, variables are presented without time indices unless a specific temporal reference is required to avoid ambiguity. These variables without time indices denote their most up-to-date values at the time of calculation. For reproducibility, the complete pseudocode for the shock, default-contagion, liquidation, and default-loss accounting procedures is provided in Section S1 of the Supplementary Materials (Algorithms S1 and S2).

Within each iteration, the financial positions of banks and firms are updated sequentially as events occur, following the rules below. In the first iteration, a random permutation of firms is generated, and firms are selected in that order until their cumulative pre-shock assets first reach or exceed a fraction ε of the total pre-shock assets of the firm sector. The realized shock share may therefore slightly exceed the target value ε and is recorded for each simulation run. The selected firms are then subjected to exogenous shocks sequentially in the same order. Exogenous shocks are imposed exclusively on the firm sector, allowing us to examine how firm-originated disturbances propagate through the credit linkages of the multilayer financial network and subsequently affect the banking sector. For each selected firm, fixed assets and cash are set to zero on its balance sheet, and its equity is adjusted accordingly to maintain the accounting identity. If a shocked firm’s equity becomes negative (i.e., $E_{j}^{f}<0$), it is deemed bankrupt and liquidated immediately, before the next shocked firm is processed, so that firms shocked later may face balance sheets already altered by these liquidations. Any bank or firm outside the initially shocked set that becomes bankrupt as a result of these liquidations is liquidated in the next iteration. Thereafter, bankruptcies generated in one iteration are liquidated in the next, and the process continues until no new bankrupt entities emerge. In each iteration after the first, banks are processed before firms, and entities within each sector are processed in ascending order of their indices. Because balance-sheet variables are updated immediately after each liquidation, earlier liquidations may affect entities processed later in the same iteration. We therefore also examine the sensitivity of the results to alternative liquidation priorities (Section 3.6.2). The liquidation process involves bankrupt entities collecting available funds and repaying their debts according to a specific priority order. The following subsections describe in detail the liquidation mechanisms for bankrupt firms and banks.

For clarity, we distinguish two failure modes. Bankruptcy triggered by negative equity is referred to as insolvency, whereas bankruptcy triggered by the exhaustion of available cash when meeting repayment obligations is referred to as illiquidity. An entity may exhibit both failure modes.

#### 2.5.1. Liquidation of Bankrupt Firms

The liquidation funds of a bankrupt firm are sourced from three channels: recovered accounts receivable, the gross liquidation value of fixed assets, and remaining cash assets. The liquidation process unfolds as follows. First, the bankrupt firm recovers outstanding accounts receivable from its debtor firms. To maintain model tractability, we assume that a debtor firm can only settle its debts using its cash assets and cannot generate additional funds by collecting its accounts receivable or liquidating fixed assets. This assumption prevents recursive debt recovery loops across firms, ensuring that liquidation cascades remain finite and well-defined within each iteration. If a debtor firm has insufficient cash assets, it transfers all its available cash assets and is then declared bankrupt due to illiquidity, entering liquidation in the next iteration. The accounts receivable recovered by bankrupt firm *j* are given by

(24) $$R_{j}^{AR}=\sum_{k=1}^{N_{f}}\min\left(L_{jk}^{ff},C_{k}^{f}\right),$$

where $L_{jk}^{ff}$ and $C_{k}^{f}$, defined in Section 2.3 and Section 2.4, represent the amount owed by firm *k* to firm *j* and the cash assets of firm *k*, respectively. Whenever a claim is processed during liquidation, the corresponding claim–liability relationship is closed after the resulting payment, which may be zero; any unrecovered amount is written off immediately and is not carried forward for recovery in subsequent iterations. Next, the bankrupt firm liquidates its fixed assets. It then pools the recovered receivables $R_{j}^{AR}$, the gross liquidation value of fixed assets $K_{j}^{f}$, and its cash assets $C_{j}^{f}$ to form a liquidation fund. To account for liquidation-related costs, such as administrative fees, employee wages, taxes, and liquidation discounts on fixed assets, we introduce the firm liquidation recovery rate $\delta_{f}$, where $0<\delta_{f}<1$. The actual funds available for debt repayment are then computed as

(25) $$F_{j}^{f}=\delta_{f}\left(R_{j}^{AR}+K_{j}^{f}+C_{j}^{f}\right).$$

Debt repayment follows a strict priority order: bank loans are repaid first, followed by interfirm debts (i.e., accounts payable). This priority order reflects the common institutional arrangement in which bank loans generally have higher repayment priority than trade credit. If the available funds $F_{j}^{f}$ are sufficient to cover all bank loans, they are repaid in full; otherwise, repayments are allocated on a pro rata basis according to each bank’s claim. The amount repaid to creditor bank *i* is

(26) $$R_{ij}^{fb}=\left\{\begin{array}{cc} L_{ij}^{bf}, & F_{j}^{f}\geq L_{j}^{fb}\\ \frac{L_{ij}^{bf}}{L_{j}^{fb}}F_{j}^{f}, & F_{j}^{f}<L_{j}^{fb} \end{array}\right.,$$

where $L_{ij}^{bf}$ and $L_{j}^{fb}$, defined in Section 2.1, represent the loan from bank *i* to firm *j* and the total bank loans of firm *j*, respectively. Upon receiving repayment, the creditor bank increases its cash assets by the repayment amount and removes the entire corresponding loan from its total loans to firms. The shortfall between the original loan amount and the repayment amount is deducted from the creditor bank’s equity as a loss to maintain the accounting identity. If the creditor bank’s equity becomes negative as a result, it is declared bankrupt due to insolvency and enters liquidation in the next iteration. The remaining funds after repayment to banks are calculated as

(27) $$F_{j}^{fr}=\max\left(F_{j}^{f}-L_{j}^{fb},0\right).$$

These funds are subsequently used to settle interfirm debts (i.e., the accounts payable of the bankrupt firm). If sufficient, these debts are paid in full; otherwise, payments are allocated on a pro rata basis according to each creditor firm’s claim. The amount paid to creditor firm $j^{\prime }$ is

(28) $$R_{j^{\prime }j}^{ff}=\left\{\begin{array}{cc} L_{j^{\prime }j}^{ff}, & F_{j}^{fr}\geq L_{j}^{AP}\\ \frac{L_{j^{\prime }j}^{ff}}{L_{j}^{AP}}F_{j}^{fr}, & F_{j}^{fr}<L_{j}^{AP} \end{array}\right.,$$

where $L_{j^{\prime }j}^{ff}$ and $L_{j}^{AP}$, defined in Section 2.3, represent the amount owed by firm *j* to firm $j^{\prime }$ and the accounts payable of firm *j*, respectively. The payment to a creditor firm may be partial or even zero, depending on the bankrupt firm’s remaining funds. Upon receiving payment, the creditor firm increases its cash assets by the payment amount and removes the entire corresponding receivable from its accounts receivable. The shortfall between the original receivable amount and the payment amount is deducted from the creditor firm’s equity as a loss to maintain the accounting identity. If the creditor firm’s equity becomes negative as a result, it is declared bankrupt due to insolvency and enters liquidation in the next iteration.

#### 2.5.2. Liquidation of Bankrupt Banks

The liquidation funds of a bankrupt bank are sourced from three channels: recovered interbank lending, recovered loans to firms, and remaining cash assets. The liquidation process unfolds as follows. First, the bankrupt bank recovers its interbank lending. As before, we assume that a debtor bank can only repay its interbank borrowing using its cash assets and cannot generate additional funds by recalling its interbank lending or loans to firms. If a debtor bank has insufficient cash assets, it transfers all its remaining cash assets and is declared bankrupt due to illiquidity, entering liquidation in the next iteration. The interbank lending recovered by bankrupt bank *i* is given by

(29) $$R_{i}^{\mathrm{IBL}}=\sum_{k=1}^{N_{b}}\min\left(L_{ik}^{bb},C_{k}^{b}\right),$$

where $L_{ik}^{bb}$ represents the interbank loan from bank *i* to bank *k* (as defined in Section 2.2), and $C_{k}^{b}$ represents the cash assets of bank *k* (as defined in Section 2.4). Next, the bankrupt bank recovers its loans to firms. Similarly, a debtor firm can only repay its bank loan using its cash assets and cannot generate additional funds by collecting its accounts receivable or liquidating fixed assets. If a debtor firm has insufficient cash assets, it transfers all its remaining cash assets and is then declared bankrupt due to illiquidity, entering liquidation in the next iteration. The loans to firms recovered by bankrupt bank *i* are given by

(30) $$R_{i}^{bf}=\sum_{k=1}^{N_{f}}\min\left(L_{ik}^{bf},C_{k}^{f}\right),$$

where $L_{ik}^{bf}$ represents the loan from bank *i* to firm *k* (as defined in Section 2.1), and $C_{k}^{f}$ represents the cash assets of firm *k* (as defined in Section 2.4). As in the liquidation of bankrupt firms, each processed claim–liability relationship is closed after settlement, and any unrecovered amount is written off immediately rather than carried forward to subsequent iterations. Bankrupt bank *i* then pools the recovered interbank lending $R_{i}^{\mathrm{IBL}}$, the recovered loans to firms $R_{i}^{bf}$, and its own cash assets $C_{i}^{b}$ to form a liquidation fund. To account for liquidation-related costs, we introduce the bank liquidation recovery rate $\delta_{b}$, where $0<\delta_{b}<1$. The actual funds available for debt repayment are then computed as

(31) $$F_{i}^{b}=\delta_{b}\left(R_{i}^{\mathrm{IBL}}+R_{i}^{bf}+C_{i}^{b}\right).$$

The allocation of these liquidation funds follows a strict priority order. The liquidation funds are first used to repay deposit liabilities. This priority order reflects the common institutional arrangement in which deposit liabilities are given priority over interbank claims. The remaining funds after deposit repayment are denoted by $F_{i}^{br}$ and calculated as

(32) $$F_{i}^{br}=\max\left(F_{i}^{b}-D_{i}^{b},0\right),$$

where $D_{i}^{b}$ represents the deposit liabilities of bank *i* (as defined in Section 2.4). These remaining funds are then used to settle interbank borrowing. If $F_{i}^{br}$ is sufficient, interbank borrowing is repaid in full; otherwise, repayments are allocated on a pro rata basis according to each creditor bank’s claim. The amount repaid to creditor bank $i^{\prime }$ is calculated as

(33) $$R_{i^{\prime }i}^{bb}=\left\{\begin{array}{cc} L_{i^{\prime }i}^{bb}, & F_{i}^{br}\geq L_{i}^{\mathrm{IBB}}\\ \frac{L_{i^{\prime }i}^{bb}}{L_{i}^{\mathrm{IBB}}}F_{i}^{br}, & F_{i}^{br}<L_{i}^{\mathrm{IBB}} \end{array}\right.,$$

where $L_{i^{\prime }i}^{bb}$ denotes the interbank loan from bank $i^{\prime }$ to bank *i*, and $L_{i}^{\mathrm{IBB}}$ represents the interbank borrowing of bank *i*, both as defined in Section 2.2. Upon receiving repayment, which may be zero if the liquidation funds are insufficient to cover deposit liabilities, the creditor bank increases its cash assets by the repayment amount and removes the entire corresponding interbank loan from its interbank lending. The shortfall between the original loan amount and the repayment amount is deducted from the creditor bank’s equity as a loss to maintain the accounting identity. If the creditor bank’s equity becomes negative as a result, it is declared bankrupt due to insolvency and enters liquidation in the next iteration.

#### 2.5.3. Quantification of Default Losses

Default loss refers to the reduction in an entity’s asset value resulting from counterparties’ failure to meet financial obligations, manifested as the unrecovered portion of the entity’s financial claims. In the financial system, such losses erode balance sheets, potentially precipitating cascading bankruptcies and risk propagation. To characterize the realized severity and distribution of losses during default contagion, we quantify default losses separately in the bank–firm, interbank, and interfirm layers.

In the bank–firm network, default losses occur when a bankrupt creditor bank fails to fully recover its loans to firms, or when a bankrupt debtor firm cannot fully repay its bank loans. For a bankrupt creditor bank *i*, the recovered amount of loans to firms $R_{i}^{bf}$ is given by Equation (30). The corresponding default loss is the unrecovered portion:

(34) $$L_{i}^{bf}=L_{i}^{bf}-R_{i}^{bf}.$$

For a bankrupt debtor firm *j*, the available liquidation funds $F_{j}^{f}$ are determined by Equation (25). Since these funds are first used to repay bank loans $L_{j}^{fb}$ (up to the full amount owed), the default loss to creditor banks is the unpaid portion:

(35) $$L_{j}^{fb}=\max\left(L_{j}^{fb}-F_{j}^{f},0\right).$$

Let $B_{t}$ and $F_{t}$ denote the sets of banks and firms liquidated during iteration *t*, respectively. The total default loss in the bank–firm network during iteration *t* is

(36) $$L_{bf}\left(t\right)=\sum_{i\in B_{t}}L_{i}^{bf}+\sum_{j\in F_{t}}L_{j}^{fb}.$$

To quantify loss severity in the bank–firm layer, we introduce the cumulative default loss ratio $l_{bf}$. Since default losses in this layer arise directly from bank loans to firms, the initial total amount of such loans provides a natural normalization baseline. This ratio is thus defined as the total default losses during the contagion process normalized by this baseline:

(37) $$l_{bf}=\frac{\sum_{t=1}^{T}L_{bf}\left(t\right)}{\sum_{i=1}^{N_{b}}L_{i}^{bf}\left(0\right)},$$

where $L_{i}^{bf}\left(0\right)$ denotes the initial loan amount extended by bank *i* to firms. The contagion process terminates at iteration *T* when no unliquidated bankrupt entities remain.

In the interbank network, default losses arise when a bankrupt creditor bank fails to fully recover its interbank lending, or when a bankrupt debtor bank cannot fully repay its interbank borrowing. For a bankrupt creditor bank *i*, the recovered interbank lending $R_{i}^{\mathrm{IBL}}$ is given by Equation (29). The resulting default loss is

(38) $$L_{i}^{\mathrm{IBL}}=L_{i}^{\mathrm{IBL}}-R_{i}^{\mathrm{IBL}}.$$

Simultaneously, bank *i* also acts as a debtor in the interbank network. As a bankrupt debtor bank, bank *i* uses the remaining liquidation funds after repaying deposit liabilities, denoted by $F_{i}^{br}$ (see Equation (32)), to repay its interbank borrowing. The default loss imposed on creditor banks is given by the repayment shortfall:

(39) $$L_{i}^{\mathrm{IBB}}=\max\left(L_{i}^{\mathrm{IBB}}-F_{i}^{br},0\right).$$

The total default loss in the interbank network during iteration *t* is

(40) $$L_{bb}\left(t\right)=\sum_{i\in B_{t}}\left(L_{i}^{\mathrm{IBL}}+L_{i}^{\mathrm{IBB}}\right).$$

To quantify loss severity in the interbank layer, we introduce the cumulative default loss ratio $l_{bb}$. Since default losses in this layer arise directly from interbank lending, this ratio normalizes the total default losses during the contagion process by the initial total amount of such lending:

(41) $$l_{bb}=\frac{\sum_{t=1}^{T}L_{bb}\left(t\right)}{\sum_{i=1}^{N_{b}}L_{i}^{\mathrm{IBL}}\left(0\right)},$$

where $L_{i}^{\mathrm{IBL}}\left(0\right)$ represents the initial value of bank *i*’s interbank lending.

In the interfirm network, default losses occur when a bankrupt creditor firm fails to fully recover its accounts receivable, or when a bankrupt debtor firm cannot fully settle its accounts payable. For a bankrupt creditor firm *j*, the recovered accounts receivable $R_{j}^{AR}$ is given by Equation (24). The associated default loss is

(42) $$L_{j}^{AR}=L_{j}^{AR}-R_{j}^{AR}.$$

Likewise, firm *j* also serves as a debtor in the interfirm network. As a bankrupt debtor firm, firm *j* uses the remaining liquidation funds after repaying bank loans, denoted by $F_{j}^{fr}$ (see Equation (27)), to settle its accounts payable. The default loss imposed on creditor firms is given by the repayment shortfall:

(43) $$L_{j}^{AP}=\max\left(L_{j}^{AP}-F_{j}^{fr},0\right).$$

The total default loss in the interfirm network during iteration *t* is

(44) $$L_{ff}\left(t\right)=\sum_{j\in F_{t}}\left(L_{j}^{AR}+L_{j}^{AP}\right).$$

To quantify loss severity in the interfirm layer, we introduce the cumulative default loss ratio $l_{ff}$. Since default losses in this layer arise directly from accounts receivable, this ratio normalizes the total default losses during the contagion process by the initial total amount of accounts receivable:

(45) $$l_{ff}=\frac{\sum_{t=1}^{T}L_{ff}\left(t\right)}{\sum_{j=1}^{N_{f}}L_{j}^{AR}\left(0\right)},$$

where $L_{j}^{AR}\left(0\right)$ denotes the initial accounts receivable of firm *j*. These layer-specific loss ratios provide ex-post measures of default-loss severity in each network layer. The interfirm loss ratio $l_{ff}$ and the interbank loss ratio $l_{bb}$ characterize losses arising within the interfirm and interbank credit layers, respectively, whereas the bank–firm loss ratio $l_{bf}$ characterizes losses arising through bank–firm credit relationships. Because these ratios are recorded for every realization, they can be evaluated conditional on the realized system state rather than only through unconditional averages; this distinction is developed in Section 2.6.1.

### 2.6. Characterization of System-Wide Collapse and Systemic Risk Measures

In nonlinear interconnected networks, system-wide collapse is an important manifestation of systemic risk. To better understand this behavior in the multilayer financial network, we examine bankruptcy fractions, susceptibility, and finite-size behavior from complementary perspectives. We further quantify systemic risk along three dimensions—collapse likelihood, shock tolerance, and financial damage—using system-wide collapse probability, collapse thresholds, and layer-specific loss ratios, respectively. The following subsections define these quantities and explain how they are estimated.

#### 2.6.1. Bankruptcy Fractions and Collapse Probabilities

For each simulation realization at a given shock intensity ε, we define the cumulative bankruptcy fractions of the bank and firm sectors as

(46) $$\rho_{b}=\frac{n_{b}}{N_{b}},\;\rho_{f}=\frac{n_{f}}{N_{f}},$$

where $n_{b}$ and $n_{f}$ denote the numbers of banks and firms, respectively, that become bankrupt at any point during the contagion process. Initial exogenous shocks are applied exclusively to firms, and $n_{f}$ includes firms rendered bankrupt directly by these shocks. Banks can become bankrupt only through subsequent endogenous contagion.

To distinguish sector-wide collapse from partial default contagion, we define the sectoral collapse indicators,

(47) $$I_{b}=1\left(\rho_{b}\geq q\right),\;I_{f}=1\left(\rho_{f}\geq q\right),$$

where 1(·) is the indicator function. Setting *q* close to unity defines sector-wide collapse as the bankruptcy of nearly all entities in the corresponding sector. Unless otherwise stated, we set q=0.99. System-wide collapse requires both sectors to collapse:

(48) $$I_{\mathrm{sys}}=I_{b}I_{f}.$$

The pair $\left(I_{b},I_{f}\right)$ identifies four mutually exclusive collapse configurations: neither sector (0,0), bank-only (1,0), firm-only (0,1), and joint collapse (1,1). Their probabilities are denoted by $P_{00}$, $P_{10}$, $P_{01}$, and $P_{11}$, respectively.

For each shock intensity, collapse probabilities are estimated from *R* independent realizations:

(49) $$P_{b}\left(\varepsilon \right)=\frac{1}{R}\sum_{r=1}^{R}I_{b}^{\left(r\right)},\;P_{f}\left(\varepsilon \right)=\frac{1}{R}\sum_{r=1}^{R}I_{f}^{\left(r\right)},$$

and

(50) $$P_{\mathrm{sys}}\left(\varepsilon \right)=\frac{1}{R}\sum_{r=1}^{R}I_{\mathrm{sys}}^{\left(r\right)}=P_{11}\left(\varepsilon \right).$$

The sectoral collapse probabilities satisfy $P_{b}=P_{10}+P_{11}$ and $P_{f}=P_{01}+P_{11}$. Bankruptcy fractions measure the extent of bankruptcy within individual realizations, whereas collapse probabilities measure how frequently the specified collapse criterion is met across realizations at a given shock intensity.

However, collapse probabilities do not capture the magnitude of the associated financial damage. The layer-specific default loss ratios defined in Section 2.5.3 therefore provide a complementary measure of financial severity. Because these ratios are recorded for each realization, we calculate their means conditional on whether system-wide collapse occurs, allowing loss severity to be compared directly between the two groups. Conditional means are reported only when the corresponding state is observed in the simulation sample.

#### 2.6.2. Collapse Thresholds and Sectoral Differences

To quantify the shock intensity associated with a specified collapse probability, we define the collapse threshold $\varepsilon_{p}^{X}$ for X∈{b,f,sys} as

(51) $$\varepsilon_{p}^{X}=\inf\left\{\varepsilon :P_{X}\left(\varepsilon \right)\geq \frac{p}{100}\right\},\;p\in \left\{10,50,90\right\}.$$

The collapse threshold is estimated at the first crossing of the specified collapse-probability level, using linear interpolation between adjacent points on the shock-intensity grid. If the specified collapse-probability level is not reached within the shock-intensity range examined in the corresponding experiment, the collapse threshold is left undefined rather than extrapolated.

We designate $\varepsilon_{50}^{\mathrm{sys}}$ as the system-wide collapse threshold. A higher $\varepsilon_{50}^{\mathrm{sys}}$ indicates that a stronger external shock is needed for the system-wide collapse probability to reach 0.5, implying greater system-wide shock tolerance. Thus, $\varepsilon_{50}^{\mathrm{sys}}$ serves as our primary measure of system-wide shock tolerance. We also report $\varepsilon_{10}^{\mathrm{sys}}$ and $\varepsilon_{90}^{\mathrm{sys}}$. The transition width of the system-wide collapse probability is defined as the shock-intensity interval between its first crossings of 0.1 and 0.9:

(52) $$W_{10\text{–}90}=\varepsilon_{90}^{\mathrm{sys}}-\varepsilon_{10}^{\mathrm{sys}}.$$

A smaller $W_{10\text{–}90}$ indicates a sharper transition, with the system-wide collapse probability rising from 0.1 to 0.9 over a narrower range of shock intensity.

To compare the shock tolerance of the bank and firm sectors, we define the difference between their collapse thresholds as

(53) $$S_{\mathrm{sector}}=\varepsilon_{50}^{b}-\varepsilon_{50}^{f}.$$

A positive value indicates that the banking sector reaches a collapse probability of 0.5 at a higher shock intensity than the firm sector, while a negative value indicates the reverse.

#### 2.6.3. Susceptibility and Finite-Size Analysis

As the number of financial entities increases, fluctuations in bankruptcy fractions tend to decrease when bankruptcies are independent, because individual-level randomness is averaged out. Correlations among bankruptcies, however, may weaken this averaging effect. To quantify fluctuations in bankruptcy fractions at a given shock intensity, we define the susceptibilities of the bank and firm sectors as

(54) $$\chi_{b}=N_{b}\left(\langle \rho_{b}^{2}\rangle -{\langle \rho_{b}\rangle }^{2}\right),\;\chi_{f}=N_{f}\left(\langle \rho_{f}^{2}\rangle -{\langle \rho_{f}\rangle }^{2}\right),$$

where 〈·〉 denotes the ensemble average. Under an independent-bankruptcy benchmark, the variance of the bankruptcy fraction typically decreases in proportion to $1/N_{x}$, leaving $\chi_{x}$ approximately constant as the sector grows. Examining how susceptibility changes with system size therefore helps assess whether, in larger systems, fluctuations in bankruptcy fractions diminish as expected under this benchmark or remain substantial.

For each sector x∈{b,f}, we identify the susceptibility peak $\chi_{x}^{\max}$ as the maximum observed on the shock-intensity grid and its location $\varepsilon_{\chi ,x}$ as the corresponding shock intensity. To characterize the width of the susceptibility peak, we use the full width at half maximum (FWHM), defined as the difference between the shock intensities at which susceptibility reaches half its peak value, $\chi_{x}^{\max}/2$, on either side of the peak. These crossing points are estimated by linear interpolation between adjacent shock-intensity grid points whose susceptibility values bracket the half-maximum level. A smaller FWHM indicates that the peak is confined to a narrower shock-intensity interval and is therefore sharper. If both crossings are not observed within the examined range, the FWHM is left undefined rather than extrapolated. We compare the peak heights, locations, and FWHMs across system sizes for the bank and firm sectors separately in Section 3.2.

## 3. Numerical Results and Criticality Analysis

This section presents the numerical analysis of the multilayer financial network under exogenous shocks. We begin with the baseline configuration, characterizing its network structure and the resulting bankruptcy fractions, susceptibility, collapse probabilities, and layer-specific financial losses. We then examine how these features change with system size through finite-size analysis. Next, matched two- and three-layer experiments assess the contribution of endogenous interfirm credit propagation. We subsequently analyze how selected balance-sheet and interfirm-network parameters affect shock tolerance and sectoral responses, followed by robustness and numerical-sensitivity analyses.

### 3.1. Baseline Network and Shock Response

#### 3.1.1. Baseline Parameter Configuration and Network Characteristics

The baseline system consists of 200 banks and 3000 firms connected through three directed credit layers: interbank lending, bank–firm lending, and interfirm trade credit. The procedures for network generation and balance-sheet construction are described in Section 2.1, Section 2.2, Section 2.3 and Section 2.4. The baseline parameter configuration is summarized in Table 3. The baseline values are used as stylized reference settings rather than as empirically calibrated estimates.

To characterize the topology generated by the baseline configuration, we analyze 150 independently generated pre-shock networks, separate from the realizations used in the contagion experiments. All links are directed from creditors to debtors. In the bank–firm layer, a bank’s out-degree is the number of firms it lends to, whereas a firm’s in-degree is the number of banks it borrows from. Table 4 reports the resulting network statistics.

In the bank–firm layer, the realized mean out-degree of banks is 33.815, and the mean in-degree of firms is 2.254, compared with the initial targets of 30 and 2, respectively. The higher realized values reflect the addition of links to ensure that every bank and firm has at least one link. Degree–asset correlations are positive in all three layers, consistent with the asset-dependent network construction. In the interbank and interfirm layers, these correlations are stronger for in-degree than for out-degree. The out–in degree correlations are close to zero in all three layers, indicating that pronounced disassortative mixing is not reproduced under the baseline configuration. The degree distributions differ both across layers and between out-degree and in-degree within each layer. Figure S1 in Section S2 of the Supplementary Materials presents their complementary cumulative distributions. These descriptive diagnostics do not establish a power-law form for the generated degree distributions. Accordingly, the networks are treated as stylized financial configurations rather than empirically calibrated replicas.

For the baseline shock experiment, the prescribed shock intensity ranges from ε=0.120 to 0.220 in increments of 0.001, with 500 independent realizations at each grid point. A new network and its associated balance sheets are generated for each realization, together with a new selection of shocked firms.

#### 3.1.2. Bimodal Bankruptcy Fractions and Susceptibility

**Figure 1:**
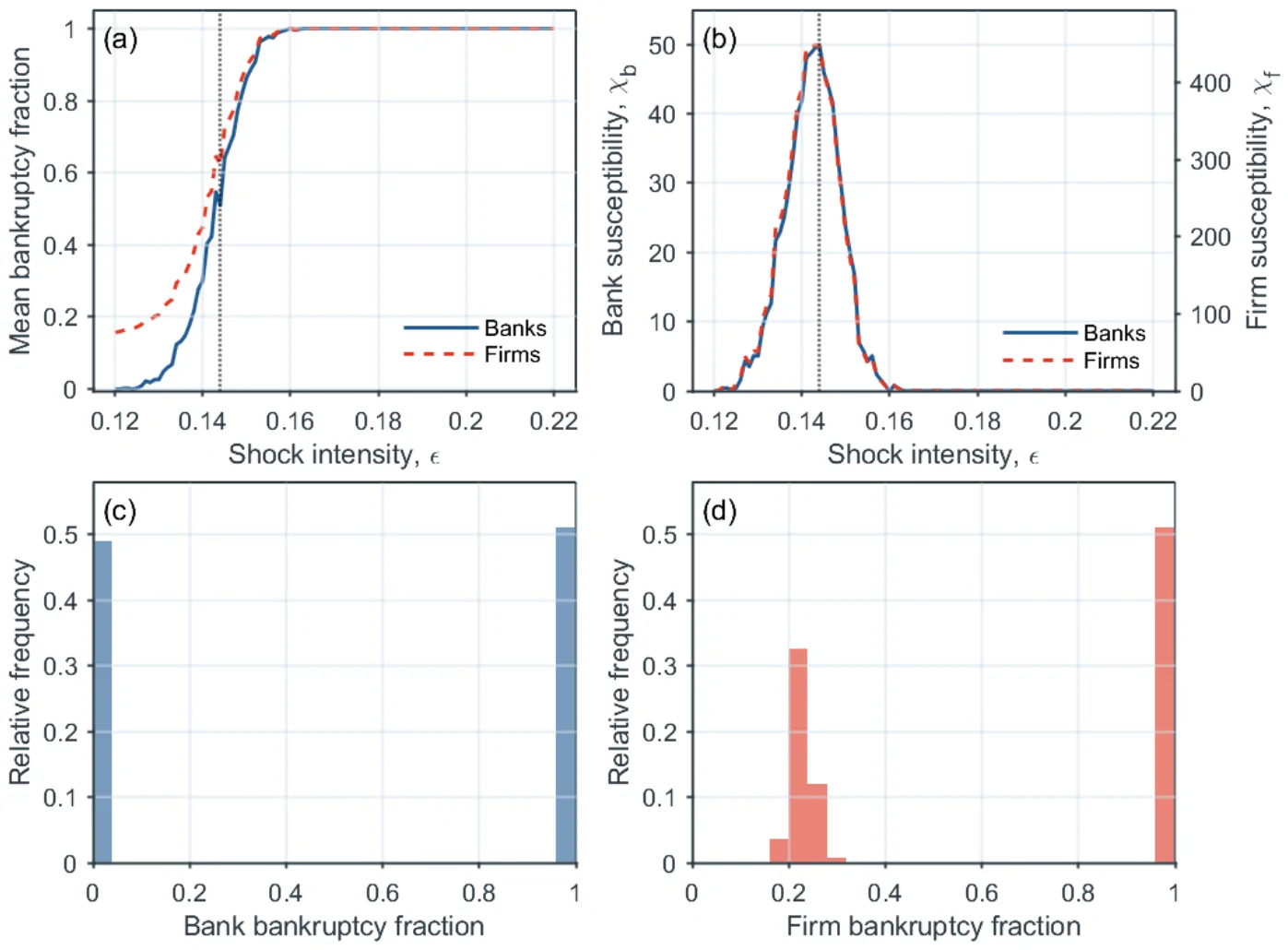
Baseline bankruptcy fractions and susceptibility. (**a**) Ensemble-averaged bankruptcy fractions of banks and firms as functions of shock intensity. (**b**) Bank and firm susceptibilities, shown against the left and right vertical axes, respectively. The dotted vertical lines in panels (**a**,**b**) mark ε=0.144, where both susceptibilities reach their maxima on the simulated shock-intensity grid and at which the distributions in panels (**c**,**d**) are evaluated. (**c**) Distribution of bank bankruptcy fractions at ε=0.144. (**d**) Distribution of firm bankruptcy fractions at the same shock intensity.

We first examine the ensemble-averaged cumulative bankruptcy fractions of banks and firms as functions of shock intensity ε. As shown in Figure 1a, both sectors exhibit a pronounced nonlinear response. At ε=0.120, the mean bankruptcy fractions are approximately 0.00025 for banks and 0.157 for firms. Both increase rapidly over a relatively narrow shock-intensity range and reach unity at ε=0.160. However, the ensemble averages alone do not reveal how bankruptcy fractions are distributed across individual realizations.

To determine where bankruptcy fractions exhibit the strongest fluctuations, we examine susceptibility. Since $\chi_{x}=N_{x}\mathrm{Var}\left(\rho_{x}\right)$ for x∈{b,f}, susceptibility provides a size-scaled measure of the variance across realizations. As shown in Figure 1b, the bank and firm susceptibilities both reach their observed maxima at ε=0.144, with $\chi_{b}^{\max}=49.935$ and $\chi_{f}^{\max}=448.784$. We therefore examine the realization-level distributions at this shock intensity.

Figure 1c,d reveal two distinct groups of realizations. In 255 of the 500 realizations, both sectors experience complete collapse, with bankruptcy fractions equal to unity. The remaining 245 realizations exhibit limited contagion, with mean bankruptcy fractions of 0.00045 for banks and 0.227 for firms. The ensemble means at this shock intensity therefore result from averaging over these two distinct groups rather than from realizations concentrated at intermediate bankruptcy fractions.

The bimodal distributions raise a further question: whether the large fluctuations identified by susceptibility arise mainly from variation within each group or from the separation between them. To quantify these two sources of variation, let *H* denote the system-wide collapse state and *L* its complement. For a given shock intensity, we define $p_{H}$ as the probability of *H* and $\mu_{H,x}$ and $\mu_{L,x}$ as the conditional mean bankruptcy fractions of sector x∈{b,f} in states *H* and *L*, respectively. The variance of the bankruptcy fraction can be decomposed as

(55) $$\mathrm{Var}\left(\rho_{x}\right)=p_{H}\mathrm{Var}\left(\rho_{x}|H\right)+\left(1-p_{H}\right)\mathrm{Var}\left(\rho_{x}|L\right)+p_{H}\left(1-p_{H}\right){\left(\mu_{H,x}-\mu_{L,x}\right)}^{2}.$$

The first two terms represent the within-state contribution, while the final term represents the between-state contribution. At ε=0.144, the between-state contribution accounts for 99.9996% of the variance in bank bankruptcy fractions and 99.8526% of the variance in firm bankruptcy fractions. Thus, the large fluctuations in bankruptcy fractions at this shock intensity arise almost entirely from the separation between the two states rather than from substantial variation within either state.

This decomposition further clarifies the origin of the susceptibility peaks. For two well-separated states, the between-state contribution contains the factor $p_{H}\left(1-p_{H}\right)$, which is maximized when the two states are equally probable. At ε=0.144, the estimated probability of system-wide collapse is $p_{H}=0.510$, placing this factor very close to its theoretical maximum. The observed susceptibility maxima therefore occur at a shock intensity where the limited-contagion and system-wide-collapse states coexist with comparable frequencies and remain strongly separated.

Overall, the analysis shows that at the observed susceptibility peak, the intermediate ensemble averages conceal a pronounced two-state structure consisting of limited contagion and system-wide collapse. Having identified this structure, Section 3.1.3 next examines how the frequency of system-wide collapse changes with shock intensity, together with the associated shock-tolerance measures and financial losses.

#### 3.1.3. Collapse Likelihood, Shock Tolerance, and Financial Damage

**Figure 2:**
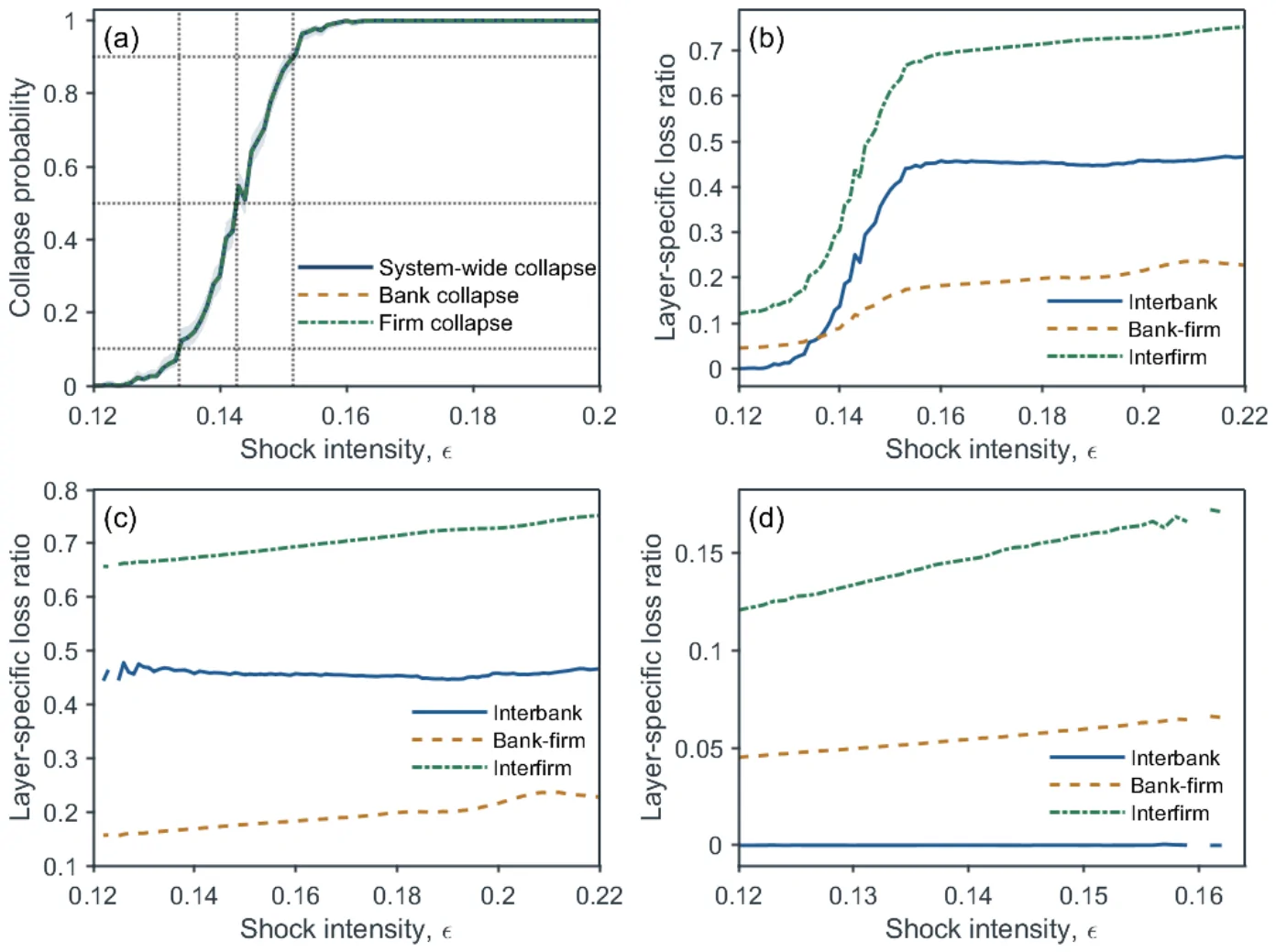
Baseline collapse probabilities and default losses. (**a**) Estimated bank, firm, and system-wide collapse probabilities as functions of shock intensity. The shaded region represents pointwise 95% Wilson confidence intervals for the system-wide collapse probability. The horizontal reference lines mark probabilities of 0.1, 0.5, and 0.9, and the vertical lines indicate the system-wide collapse thresholds $\varepsilon_{10}^{\mathrm{sys}}$, $\varepsilon_{50}^{\mathrm{sys}}$, and $\varepsilon_{90}^{\mathrm{sys}}$. (**b**) Unconditional mean default loss ratios in the interbank (BB), bank–firm (BF), and interfirm (FF) layers. (**c**,**d**) Mean default loss ratios conditional on system-wide collapse and on the absence of system-wide collapse, respectively. Conditional means are shown only at shock intensities where the corresponding state is observed among the 500 realizations.

The two-state pattern identified in Section 3.1.2 motivates a quantitative characterization of the baseline system along three complementary dimensions: collapse likelihood, shock tolerance, and financial damage. Collapse likelihood is measured by the estimated collapse probabilities, shock tolerance by the collapse thresholds, and financial damage by the layer-specific default loss ratios. We first examine how the probability of system-wide collapse changes with shock intensity. As defined in Section 2.6, system-wide collapse requires the bankruptcy fractions of both banks and firms to reach or exceed q=0.99. Figure 2a shows that the estimated probability of system-wide collapse, $P_{\mathrm{sys}}\left(\varepsilon \right)$, increases rapidly over a narrow shock-intensity range. It rises from 0.300 at ε=0.140 to 0.864 at ε=0.150. At ε=0.160, all 500 realizations satisfy the system-wide collapse criterion, giving an estimated collapse probability of unity. The bank, firm, and system-wide collapse probability curves nearly coincide. Across all 50,500 baseline realizations, only two exhibit sectorally asymmetric collapse: firms meet the collapse criterion while banks do not. No bank-only collapse is observed. Thus, in almost every baseline realization, either both sectors meet the collapse criterion or neither does.

Table 5 reports the estimated collapse thresholds and their bootstrap confidence intervals. These thresholds quantify shock tolerance by indicating the shock intensity required for the collapse probability to reach a specified level; higher thresholds therefore correspond to greater shock tolerance. The system-wide collapse threshold is $\varepsilon_{50}^{\mathrm{sys}}=0.142629$, with a 95% confidence interval of [0.142358,0.142956]. The collapse thresholds $\varepsilon_{10}^{\mathrm{sys}}$ and $\varepsilon_{90}^{\mathrm{sys}}$ are 0.133571 and 0.151600, respectively, giving $W_{10\text{–}90}=0.018029$. Thus, the baseline system reaches a 50% probability of system-wide collapse at a shock intensity of approximately 0.143, while the transition from a 10% to a 90% system-wide collapse probability occurs over a shock-intensity interval of about 0.018. The estimated collapse thresholds for banks, firms, and the system as a whole coincide at the probability levels of 0.1, 0.5, and 0.9, giving $S_{\mathrm{sector}}=0$. This coincidence indicates no sectoral difference in shock tolerance as measured by the collapse thresholds under the baseline configuration.

Collapse probability characterizes collapse likelihood but does not quantify the severity of the associated financial damage. We therefore use the layer-specific default loss ratios to measure financial damage across the three credit layers. Figure 2b reports the unconditional mean default loss ratios in the three credit layers, while panels (c) and (d) show the mean loss ratios conditional on whether system-wide collapse occurs. At ε=0.144, the mean interbank loss ratio is 0.4593 among system-wide-collapse realizations, compared with approximately 0.00003 among realizations without system-wide collapse. The corresponding ratios are 0.1717 and 0.0564 in the bank–firm layer and 0.6770 and 0.1529 in the interfirm layer. The near absence of interbank losses in realizations without system-wide collapse is consistent with the very low bank bankruptcy fraction reported in Section 3.1.2. However, bank–firm and interfirm losses remain present even when system-wide collapse does not occur, indicating that financial damage is not confined to realizations that meet the system-wide collapse criterion.

Loss severity can also differ between shock intensities with the same estimated system-wide collapse probability. At both ε=0.160 and 0.220, all 500 realizations satisfy the system-wide collapse criterion. Nevertheless, the mean bank–firm loss ratio increases from 0.1829 to 0.2275 between these two shock intensities, while the mean interfirm loss ratio increases from 0.6938 to 0.7520. The mean interbank loss ratio changes only slightly over the same interval. Thus, the estimated system-wide collapse probability alone does not capture differences in financial damage once it has saturated at unity.

Together, these results show that collapse likelihood, shock tolerance, and financial damage capture distinct aspects of the baseline system’s response. We next examine how collapse behavior and susceptibility vary with system size.

### 3.2. Finite-Size Dependence of Collapse Behavior

The baseline analysis reveals a pronounced two-state structure, a rapid change in system-wide collapse probability, and sharp susceptibility peaks. We now examine how these features change with system size. Five sizes are considered, with $N_{b}\in \left\{150,200,250,300,400\right\}$ and $N_{f}=15N_{b}$. To reduce the confounding effect of changes in network connectivity, the interbank and interfirm link-formation parameters, $d_{b}$ and $d_{f}$, are calibrated separately at each size to maintain approximately constant realized mean degrees. A pilot scan with 20 realizations per shock intensity is used to identify the relevant shock range for each size. The subsequent formal experiments use 500 independent realizations per shock intensity, with a grid spacing of 0.001. The formal shock ranges, ordered by increasing $N_{b}$, are [0.117,0.168], [0.116,0.167], [0.115,0.165], [0.116,0.166], and [0.117,0.167], respectively.

Table 6 summarizes the calibrated network parameters and finite-size results. The target mean degrees are 9.339 for the interbank layer and 94.136 for the interfirm layer. Across the five sizes, the measured mean degrees range from 9.336 to 9.437 and from 93.563 to 95.042, respectively. Their maximum absolute deviations from the targets are approximately 1.05% and 0.96%. The calibration therefore substantially reduces variation in mean degree across system sizes, although the realized connectivity is not identical.

**Figure 3:**
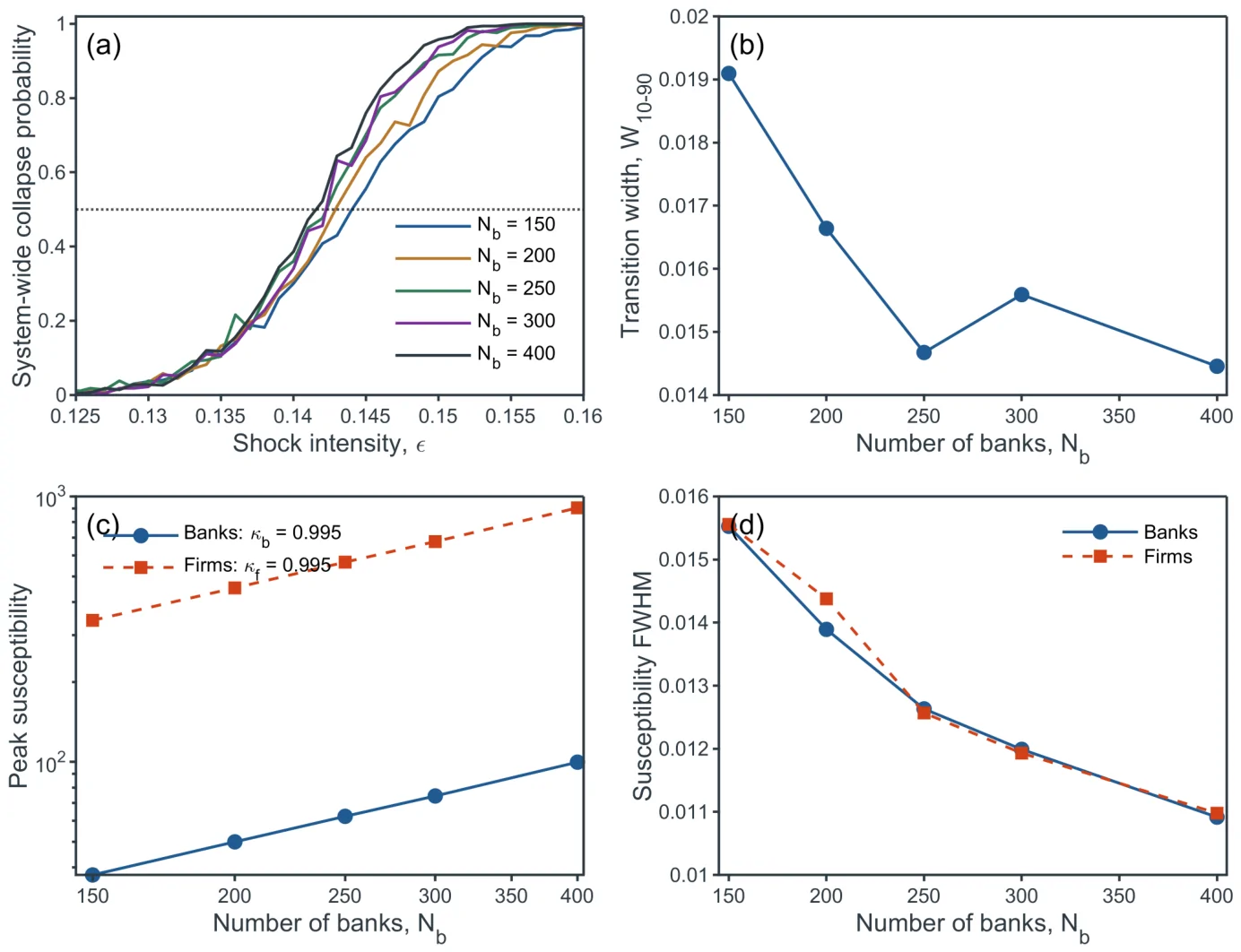
Degree-controlled finite-size analysis of the three-layer system, with $N_{f}=15N_{b}$. (**a**) Estimated system-wide collapse probabilities for five system sizes. (**b**) Transition width $W_{10\text{–}90}$, defined as the shock-intensity interval between the first crossings of 0.1 and 0.9 by the system-wide collapse probability. (**c**) Observed susceptibility peak heights as functions of $N_{b}$ on logarithmic axes; because $N_{f}=15N_{b}$, this constant rescaling does not change the fitted firm-sector exponent. The legend reports the exponents $\kappa_{b}$ and $\kappa_{f}$ obtained from log–log regression. (**d**) Full widths at half maximum (FWHM) of the bank and firm susceptibility peaks. Susceptibility peaks are identified on the observed shock grid, while $W_{10\text{–}90}$ and the FWHM values are calculated using linear interpolation where required. All results are obtained from the independent degree-controlled finite-size experiments.

Figure 3a shows that the estimated system-wide collapse probabilities rise rapidly with shock intensity at all five system sizes. The system-wide collapse threshold $\varepsilon_{50}^{\mathrm{sys}}$ changes only modestly, decreasing from 0.144034 at $N_{b}=150$ to 0.141560 at $N_{b}=400$. By contrast, the transition width $W_{10\text{–}90}$ decreases overall from 0.019096 to 0.014455, as shown in Figure 3b. The decrease is not strictly monotonic: $W_{10\text{–}90}$ increases from 0.014673 at $N_{b}=250$ to 0.015590 at $N_{b}=300$. Thus, the system-wide collapse-probability transition shifts only slightly in shock intensity but generally occurs over a narrower shock-intensity range as system size increases.

The susceptibility peak heights exhibit a stronger dependence on system size. As shown in Figure 3c, the banking-sector peak increases from 37.464 at $N_{b}=150$ to 99.681 at $N_{b}=400$, while the firm-sector peak increases from 341.317 at $N_{f}=2250$ to 905.816 at $N_{f}=6000$. To quantify this dependence, we fit the empirical relation

(56) $$\chi_{s}^{\max}\left(N_{s}\right)\propto N_{s}^{\kappa_{s}},\;s\in \left\{b,f\right\}.$$

Log–log regressions of the observed grid-based peak heights yield $\kappa_{b}=0.9954$ and $\kappa_{f}=0.9945$, with coefficients of determination exceeding 0.9999. Their respective 95% bootstrap confidence intervals are [0.9910,1.0064] and [0.9818,1.0070]. Thus, within the five sizes examined, the susceptibility peaks grow approximately linearly with the size of the corresponding sector.

The susceptibility peaks also become narrower as system size increases. Figure 3d shows that the banking-sector FWHM decreases from 0.01553 at $N_{b}=150$ to 0.01092 at $N_{b}=400$, while the firm-sector FWHM decreases from 0.01556 to 0.01098. The shock intensities at which the susceptibility peaks occur also change only slightly, remaining within ε∈[0.142,0.144] for banks and ε∈[0.141,0.144] for firms. Hence, increasing system size is associated primarily with higher and narrower susceptibility peaks rather than a substantial shift in their positions.

The two-state structure identified in Section 3.1.2 also persists across the finite-size experiments. At the sector-specific susceptibility peaks, the between-state component of the variance accounts for more than 99.999% of banking-sector variance and approximately 99.86–99.89% of firm-sector variance. Using the two-state notation introduced in Section 3.1.2, the between-state contribution to susceptibility is

(57) $$\chi_{s,\mathrm{between}}=N_{s}p_{H}\left(1-p_{H}\right){\left(\mu_{H,s}-\mu_{L,s}\right)}^{2}.$$

This expression shows that susceptibility can grow approximately proportionally to $N_{s}$ when the two states remain well separated and neither state becomes rare near the peak. The dominance of the between-state variance component in the simulations therefore provides an explanation for the observed near-linear growth of the susceptibility peaks.

Overall, as system size increases, the location of the system-wide collapse-probability transition changes only modestly, its width generally narrows, and the susceptibility peaks become higher and narrower. Together with the persistence of the two-state structure, these results provide consistent finite-size evidence of phase-transition-like collapse behavior. The following section uses matched two- and three-layer models to examine the contribution of endogenous interfirm credit propagation to collapse behavior.

### 3.3. Contribution of Endogenous Interfirm Credit Propagation: Matched Model Comparisons

The preceding analysis characterizes collapse behavior in the three-layer system but does not isolate the contribution of endogenous interfirm credit propagation. To examine this contribution, we construct two-layer counterfactuals that retain the interbank and bank–firm networks while replacing endogenous interfirm settlements with external recovery arrangements. Accounts receivable and accounts payable arising from interfirm trade credit remain on firms’ initial balance sheets, but their settlement no longer transmits payments or losses between firms. When a firm goes bankrupt, its accounts receivable are recovered externally rather than through repayments from other firms. Within each simulation pair, the three-layer and two-layer models share the same initial network realization, balance sheets, shocked firms, and realized shock intensity.

Removing endogenous interfirm settlement also changes the recovery of accounts receivable during firm liquidation. To reduce the confounding effect of this change, we consider two recovery-matching methods. Under both methods, a bankrupt firm externally recovers a fixed fraction of the face value of its accounts receivable. This external recovery rate is common to all firms and realizations at a given shock intensity but may vary across shock intensities. The two methods differ in the aggregate recovery quantity they seek to reproduce.

Under recovery-rate matching, the external recovery rate is chosen to match the aggregate recovery rate of the three-layer model. At each shock intensity ε, we calculate the total recovered amount of accounts receivable, $R_{3L}\left(\varepsilon \right)$, and their total face value, $F_{3L}\left(\varepsilon \right)$, across the 500 three-layer realizations. The external recovery rate is then

(58) $$r_{\mathrm{rate}}\left(\varepsilon \right)=\frac{R_{3L}\left(\varepsilon \right)}{F_{3L}\left(\varepsilon \right)}.$$

The resulting rate is applied in the two-layer model.

Recovery-amount matching instead targets the average recovered amount per bankrupt firm in the three-layer model. At each shock intensity, the target is

(59) $$A_{3L}\left(\varepsilon \right)=\frac{R_{3L}\left(\varepsilon \right)}{N_{3L}\left(\varepsilon \right)},$$

where $N_{3L}\left(\varepsilon \right)$ is the total number of bankrupt firms across the 500 three-layer realizations. We then use the recovery-rate-matched two-layer model to calculate the average face value of accounts receivable per bankrupt firm:

(60) $$B_{2L,\mathrm{rate}}\left(\varepsilon \right)=\frac{F_{2L,\mathrm{rate}}\left(\varepsilon \right)}{N_{2L,\mathrm{rate}}\left(\varepsilon \right)},$$

where $F_{2L,\mathrm{rate}}\left(\varepsilon \right)$ is the total face value of accounts receivable held by bankrupt firms, and $N_{2L,\mathrm{rate}}\left(\varepsilon \right)$ is the total number of bankrupt firms, both aggregated across the 500 recovery-rate-matched two-layer realizations. The external recovery rate is then set to

(61) $$r_{\mathrm{amount}}\left(\varepsilon \right)=\min\left\{1,\;\frac{A_{3L}\left(\varepsilon \right)}{B_{2L,\mathrm{rate}}\left(\varepsilon \right)}\right\}.$$

The two-layer model is subsequently rerun using this rate and the same firm-level liquidation rule as under recovery-rate matching. Because changing the recovery rate can alter subsequent bankruptcies, the achieved average recovered amount need not exactly equal the three-layer target.

**Figure 4:**
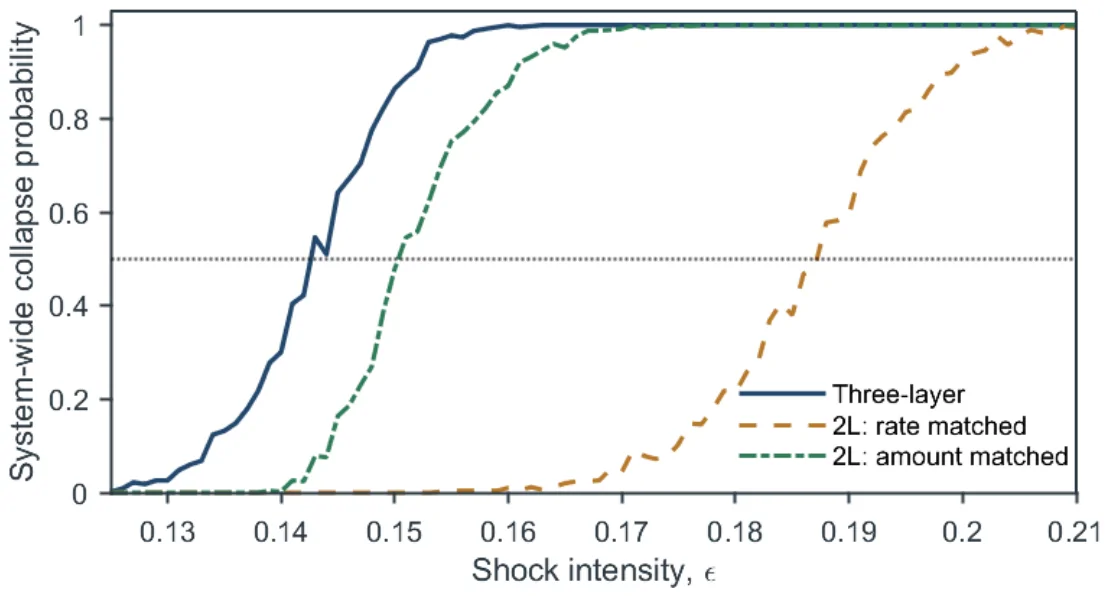
Matched comparisons between the three-layer model and two two-layer counterfactuals. The curves show estimated system-wide collapse probabilities for the three-layer model and the two two-layer counterfactuals under recovery-rate and recovery-amount matching, using paired initial conditions and shocks. The black dotted horizontal line indicates a system-wide collapse probability of 0.5; the corresponding system-wide collapse thresholds are reported in Table 7.

Figure 4 compares the resulting system-wide collapse probabilities. At ε=0.144, the estimated system-wide collapse probabilities are 0.510 in the three-layer model, 0 under recovery-rate matching, and 0.076 under recovery-amount matching, corresponding to 255, 0, and 38 system-wide collapse events, respectively, out of 500 realizations per model. The recovery-rate-matched curve is shifted farther toward higher shock intensities than the recovery-amount-matched curve.

Table 7 reports the system-wide collapse thresholds and transition widths for the three models. We define the collapse-threshold difference for each matching method as

(62) $$\Delta \varepsilon_{50}^{m}=\varepsilon_{50}^{2L,m}-\varepsilon_{50}^{3L},\;m\in \left\{\mathrm{rate},\mathrm{amount}\right\},$$

where the thresholds refer to system-wide collapse. The system-wide collapse threshold is 0.142629 in the three-layer model, compared with 0.187170 under recovery-rate matching and 0.150324 under recovery-amount matching. The corresponding threshold differences are 0.044541 and 0.007694, with 95% paired-bootstrap confidence intervals of [0.043204,0.044975] and [0.006999,0.008248], respectively. Thus, both matching methods yield higher system-wide collapse thresholds in the two-layer counterfactuals, although the magnitude of the increase differs substantially between the two methods.

The two-state structure identified in the three-layer model also persists in both two-layer counterfactuals. At the sector-specific susceptibility peaks, the between-state component accounts for more than 99.6% of the variance in both sectors under either matching method. Thus, removing endogenous interfirm credit propagation does not eliminate the pronounced separation between the two states.

The difference in threshold shifts between the two matching methods reflects the fact that they preserve different aspects of recovery in the three-layer model. Recovery-rate matching uses a single unconditional aggregate recovery rate at each shock intensity and therefore does not preserve the variation in recovery rates associated with different realized bankruptcy fractions in the three-layer model. At ε=0.144, the aggregate recovery rates in the three-layer model are approximately 0.535 in state *H* and 0.992 in state *L*, whereas the unconditional rate used for matching is 0.642. Recovery-amount matching instead targets the average recovered amount per bankrupt firm. Across the 16 shock-grid points from ε=0.145 to 0.160, the maximum absolute relative deviation from the three-layer recovery-amount target is 4.05%, and the median absolute relative deviation is 1.62%. These differences in what is preserved by the two matching methods are consistent with the substantially different threshold shifts observed in the two counterfactuals.

The effect on transition width differs between the two matching methods. Under recovery-rate matching, $W_{10\text{–}90}$ increases from 0.018029 in the three-layer model to 0.024214 in the two-layer model, giving a difference of 0.006186 with a 95% paired-bootstrap confidence interval of [0.004270,0.010962]. Under recovery-amount matching, the transition width decreases to 0.016327, giving a difference of −0.001701 with a corresponding confidence interval of [−0.002625,−0.000118]. Thus, unlike the system-wide collapse threshold, the direction of the change in transition width depends on the recovery-matching method.

These comparisons characterize differences between the specified model arrangements rather than a pure topological effect of the interfirm layer. Although the initial conditions and shocks are paired, replacing endogenous interfirm settlements also changes subsequent repayment and recovery dynamics. Neither matching method reproduces the full realization-level distribution of recoveries and financial flows. The paired-bootstrap confidence intervals treat the calibrated external recovery rates as fixed and therefore do not include the additional uncertainty associated with estimating these rates.

Overall, removing endogenous interfirm credit propagation raises the system-wide collapse threshold under both recovery-matching methods, although the magnitude of the increase depends substantially on how recovery is matched. The direction of the change in transition width is also matching-dependent, whereas the two-state structure persists in both two-layer counterfactuals. The following section examines how changes in balance-sheet composition affect collapse behavior in the three-layer system.

### 3.4. Effects of Balance-Sheet Composition

**Figure 5:**
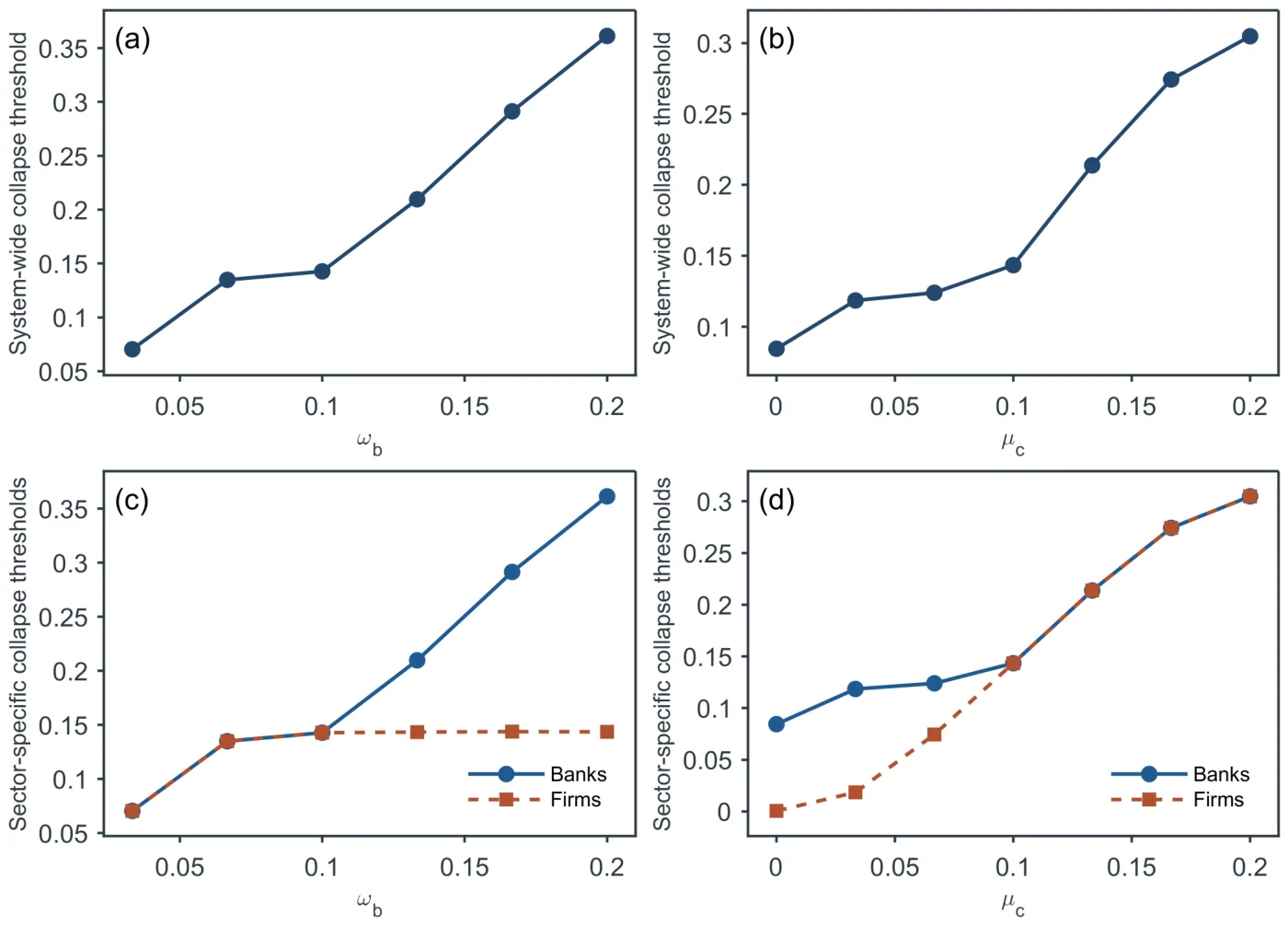
Effects of balance-sheet composition on collapse thresholds. (**a**,**b**) System-wide collapse thresholds under changes in the bank equity share $\omega_{b}$ and firm cash share $\mu_{c}$, respectively. Circles show point estimates; uncapped vertical lines indicate 95% bootstrap confidence intervals. The intervals are very narrow relative to the vertical-axis scale and may be difficult to distinguish from the circles. (**c**,**d**) Bank- and firm-sector collapse thresholds under changes in $\omega_{b}$ and $\mu_{c}$, respectively. The degenerate configuration $\omega_{b}=0$ is excluded from panels (**a**,**c**) and from quantitative threshold comparisons. At $\mu_{c}=0$, the firm-sector threshold is estimated by interpolation between the first two shock-grid points and is therefore treated as a boundary estimate.

We next examine how two aspects of balance-sheet composition affect shock tolerance: the share of equity in total assets for banks, $\omega_{b}$, and the share of cash in total assets for firms, $\mu_{c}$. Bank equity provides a buffer against losses, whereas firm cash supports the fulfillment of immediate repayment obligations. Figure 5 summarizes the effects of these two parameters on system-wide and sector-specific collapse thresholds.

#### 3.4.1. Bank Capitalization

Bank capitalization is measured by $\omega_{b}$, the share of equity in total assets. We vary $\omega_{b}$ over seven equally spaced values from 0 to 0.20, holding the remaining model parameters at their baseline settings. Under the accounting identities in Section 2.4, increasing $\omega_{b}$ raises bank equity while reducing residual deposit liabilities for a given asset size. The experiment therefore captures a change in both equity buffers and liability composition. The configuration $\omega_{b}=0$ is a degenerate boundary case and is excluded from the following threshold comparisons.

Figure 5a shows that the system-wide collapse threshold increases with bank capitalization. It rises from 0.070386 at $\omega_{b}=0.0333$ to 0.142700 at the baseline value of 0.10 and reaches 0.361278 at $\omega_{b}=0.20$. The increase is uneven, with relatively little change between $\omega_{b}=0.0667$ and 0.10 and larger increases at higher capitalization levels. Thus, higher bank capitalization is associated with greater system-wide shock tolerance under the specified balance-sheet adjustments.

The sector-specific thresholds show that this increase in system-wide shock tolerance does not reflect comparable changes in both sectors. As shown in Figure 5c, the bank and firm thresholds coincide for the nondegenerate configurations with $\omega_{b}\leq 0.10$. At $\omega_{b}=0.1333$, however, the bank threshold rises to 0.209645 while the firm threshold remains at 0.143273, giving $S_{\mathrm{sector}}=0.066372$. At $\omega_{b}=0.20$, the separation reaches 0.217761, with a bank threshold of 0.361278 and a firm threshold of 0.143517. The system-wide threshold coincides with the bank threshold throughout the investigated configurations. Hence, at higher capitalization levels, the increase in system-wide shock tolerance primarily reflects a higher banking-sector threshold rather than a comparable increase in firm-sector shock tolerance.

#### 3.4.2. Firm Liquidity

Firm liquidity is measured by $\mu_{c}$, the share of cash in total assets. We vary $\mu_{c}$ over seven equally spaced values from 0 to 0.20, holding the remaining model parameters at their baseline settings. Under the accounting identities in Section 2.4, increasing $\mu_{c}$ raises firms’ initial cash holdings while reducing their residual fixed assets. The experiment therefore represents a reallocation of firm assets toward cash rather than an isolated increase in liquidity with other balance-sheet items unchanged.

Figure 5b shows that the system-wide collapse threshold increases across the investigated liquidity range. It rises from 0.084400 at $\mu_{c}=0$ to 0.143355 at the baseline value of 0.10 and reaches 0.304800 at $\mu_{c}=0.20$. The increase is not uniform: the threshold changes only from 0.118444 to 0.123889 between $\mu_{c}=0.0333$ and 0.0667, followed by larger increases over parts of the higher-liquidity range. Thus, greater initial cash holdings are associated with higher system-wide shock tolerance under the specified asset reallocation.

The sector-specific thresholds reveal a different pattern from that observed for bank capitalization. As shown in Figure 5d, at $\mu_{c}=0.0333$, the firm-sector threshold is 0.018400, substantially below the banking-sector threshold of 0.118444, giving $S_{\mathrm{sector}}=0.100044$. The separation decreases to 0.049444 at $\mu_{c}=0.0667$, and from the baseline value of $\mu_{c}=0.10$ onward, the estimated thresholds of the two sectors coincide. At $\mu_{c}=0$, the firm-sector threshold is a boundary estimate obtained by interpolation between the first two shock-grid points and is excluded from the quantitative comparison of sectoral threshold separation. Thus, low firm liquidity is associated with a substantially lower firm-sector threshold, and this separation disappears from $\mu_{c}=0.10$ onward.

Overall, both higher bank capitalization and higher firm cash shares are associated with greater system-wide shock tolerance, but their sectoral effects differ. At high capitalization levels, the banking-sector threshold continues to increase while the firm-sector threshold changes little. At low firm liquidity, by contrast, the firm-sector threshold lies substantially below the banking-sector threshold, with the two thresholds coinciding from $\mu_{c}=0.10$ onward.

### 3.5. Effects of Interfirm Link Formation and Credit Exposure

**Figure 6:**
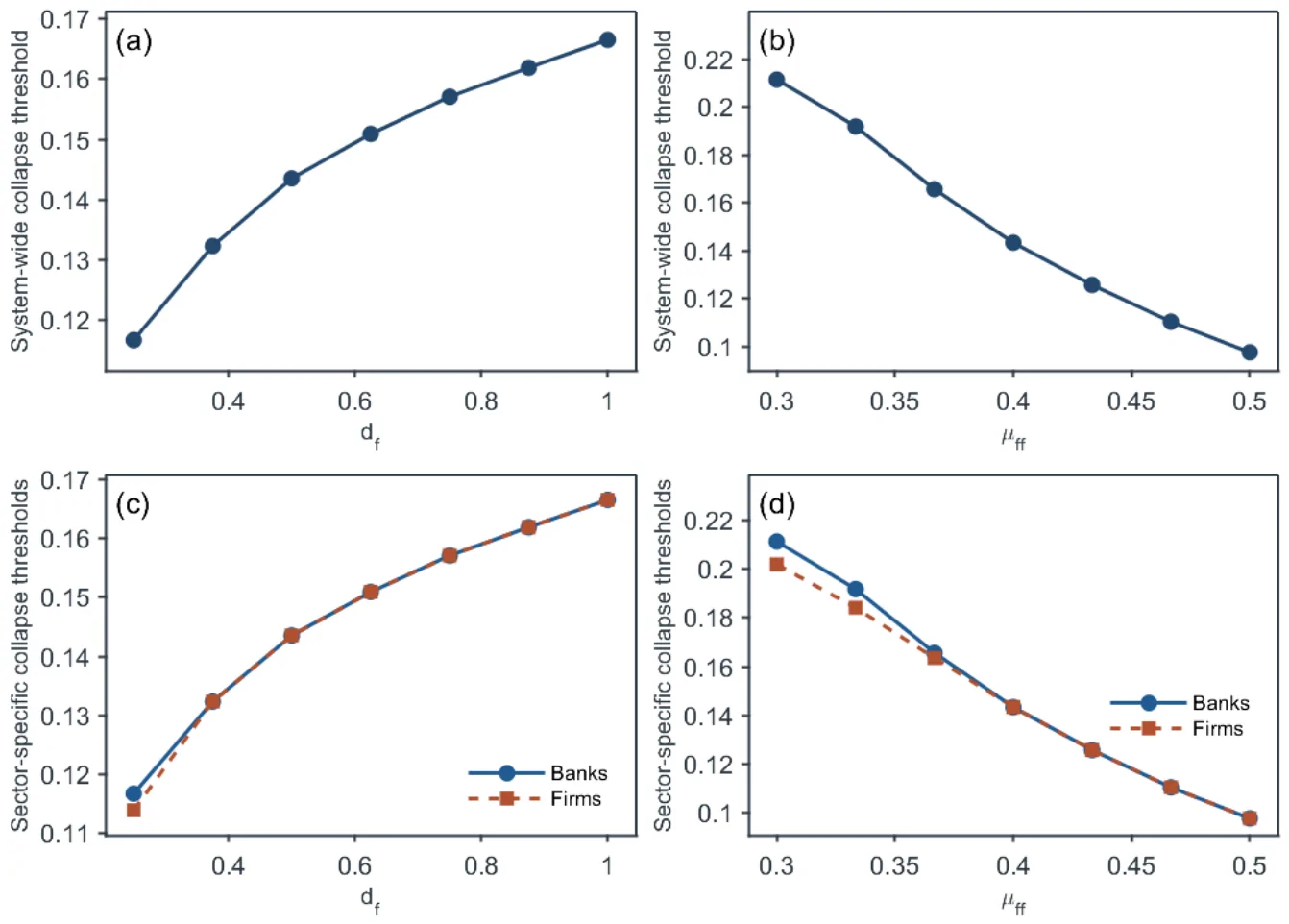
Effects of interfirm link formation and credit exposure on collapse thresholds. (**a**,**b**) System-wide collapse thresholds under changes in the interfirm link-formation parameter $d_{f}$ and the share of accounts payable in firms’ total assets $\mu_{ff}$, respectively. Circles show point estimates; uncapped vertical lines indicate 95% bootstrap confidence intervals. The intervals are very narrow relative to the vertical-axis scale and may be difficult to distinguish from the circles. (**c**,**d**) Bank- and firm-sector collapse thresholds under changes in $d_{f}$ and $\mu_{ff}$, respectively.

We next examine two aspects of the interfirm credit layer: link formation, governed by the parameter $d_{f}$, and credit exposure, governed by $\mu_{ff}$, the share of accounts payable in firms’ total assets. We vary these parameters separately to examine their effects on system-wide and sector-specific shock tolerance. Figure 6 summarizes the resulting collapse thresholds.

#### 3.5.1. Interfirm Link Formation

The parameter $d_{f}$ governs interfirm link formation. We vary $d_{f}$ over seven values from 0.25 to 1.00, holding the remaining model parameters at their baseline settings. Increasing $d_{f}$ produces a denser interfirm network and changes counterparties and the allocation of interfirm exposures, thereby affecting firms’ accounts receivable and residual fixed assets. The mean in-degree and mean out-degree of the interfirm network are identical and increase from 47.05 at $d_{f}=0.25$ to 94.47 at the baseline value of 0.50, reaching 189.33 at $d_{f}=1$. The measured mean degrees in the interbank and bank–firm layers remain approximately stable. Accordingly, this experiment should be interpreted as a change in the interfirm network configuration rather than as isolating a pure topological effect.

Figure 6a shows that the system-wide collapse threshold increases monotonically as $d_{f}$ increases. It rises from 0.116714 at $d_{f}=0.25$ to 0.143545 at the baseline value of 0.50 and reaches 0.166556 at $d_{f}=1$. The increments in the estimated threshold become progressively smaller as $d_{f}$ increases. Thus, a higher $d_{f}$, which produces a denser interfirm network, is associated with greater system-wide shock tolerance under the specified network-generation and balance-sheet rules.

The sector-specific thresholds remain closely aligned. As shown in Figure 6c, at $d_{f}=0.25$, the banking-sector threshold is 0.116714, compared with 0.113904 for the firm sector, giving $S_{\mathrm{sector}}=0.002810$. The estimated thresholds coincide at the remaining six parameter values. Thus, unlike the pronounced sectoral separation observed for some balance-sheet configurations in Section 3.4, changes in $d_{f}$ shift the bank- and firm-sector thresholds toward higher shock intensities without producing substantial separation between them.

#### 3.5.2. Interfirm Credit Exposure

The parameter $\mu_{ff}$ specifies the share of accounts payable in firms’ total assets. We vary $\mu_{ff}$ over seven values from 0.30 to 0.50, holding the remaining model parameters at their baseline settings. Increasing $\mu_{ff}$ raises firms’ accounts payable and, consequently, the corresponding accounts receivable of their suppliers. Under the accounting identities in Section 2.4, higher accounts payable reduce residual firm equity, while higher accounts receivable reduce residual fixed assets. The experiment therefore captures a combined change in trade credit exposure and balance-sheet composition.

In contrast to the $d_{f}$ experiment, increasing trade credit exposure shifts the system-wide collapse threshold toward lower shock intensities. As shown in Figure 6b, the threshold decreases monotonically from 0.211394 at $\mu_{ff}=0.30$ to 0.143333 at the baseline value of 0.40 and reaches 0.097600 at $\mu_{ff}=0.50$. Thus, higher trade credit exposure is associated with lower system-wide shock tolerance under the specified accounting and contagion rules.

The sector-specific thresholds exhibit a modest separation at lower exposure levels. As shown in Figure 6d, at $\mu_{ff}=0.30$, the firm-sector threshold is 0.202000, compared with 0.211394 for the banking sector, giving $S_{\mathrm{sector}}=0.009394$. The separation decreases to 0.007665 at $\mu_{ff}=0.3333$ and 0.002130 at $\mu_{ff}=0.3667$. From the baseline value of $\mu_{ff}=0.40$ onward, the estimated thresholds of the two sectors coincide. Thus, the modest sectoral separation observed at low exposure levels disappears as $\mu_{ff}$ reaches its baseline value.

Overall, the two parameter changes are associated with opposite movements in system-wide shock tolerance under the specified model configurations. Increasing $d_{f}$, which produces a denser interfirm network, is associated with a higher system-wide collapse threshold, whereas increasing trade credit exposure is associated with a lower threshold. In both experiments, the bank- and firm-sector thresholds remain more closely aligned than in the balance-sheet configurations examined in Section 3.4.

### 3.6. Robustness and Numerical Sensitivity

We examine the robustness of the main results to two methodological choices: the bankruptcy-fraction cutoff used to define sectoral collapse and the order in which bankrupt entities are liquidated during contagion. We also report the feasibility of the generated configurations in the baseline experiment. Additional sensitivity analysis with respect to bank and firm liquidation recovery rates is reported in Section S3 of the Supplementary Materials.

#### 3.6.1. Sensitivity to the Collapse Criterion

The preceding analysis defines sectoral collapse using a bankruptcy-fraction cutoff of q=0.99. We assess sensitivity to this choice by recalculating collapse classifications using q=0.90 and q=0.95, without rerunning the underlying simulations. In the baseline three-layer experiment, none of the 50,500 realizations changes its system-wide-collapse classification. Consequently, the estimated system-wide collapse-probability curve, collapse threshold, and $W_{10\text{–}90}$ are identical under the three cutoffs.

The system-wide-collapse classifications also remain unchanged across all simulated shock intensities in the five degree-controlled finite-size experiments. In the matched comparisons of Section 3.3, the paired system-wide-collapse classifications are likewise unchanged under both recovery-rate matching and recovery-amount matching. Thus, over the tested range of *q*, the system-wide-collapse classifications and the corresponding threshold-based results are insensitive to the bankruptcy-fraction cutoff.

#### 3.6.2. Sensitivity to Liquidation Order

The contagion algorithm liquidates bankrupt entities sequentially, with each liquidation updating the financial positions of other entities. Individual outcomes may therefore depend on liquidation order. Under the baseline convention, banks are processed before firms in each contagion round after the initial shocks, and entities within each sector are processed in ascending order of their indices. We examine four alternative liquidation priorities: random order, larger entities first, smaller entities first, and firms before banks. Entity size is measured by initial total assets. Each alternative uses the same initial financial configuration and shocked firms as its baseline counterpart, while the initial shock sequence is unchanged. Only the liquidation order in subsequent contagion rounds is varied.

The experiment covers 16 shock intensities between 0.132 and 0.154, with 500 paired realizations at each intensity. A reproduction test confirms that the baseline implementation yields identical results. Table 8 reports the estimated system-wide collapse thresholds together with the proportion of paired realizations whose system-wide-collapse classification differs from that under the baseline order.

The alternative liquidation orders produce only small changes in the estimated system-wide collapse threshold. The largest difference is 0.000113 under random priority, with a 95% paired-bootstrap confidence interval of [−0.000010,0.000430]. The corresponding confidence intervals for the larger-first and smaller-first conventions also include zero, while the firms-first convention yields the same estimated threshold as the baseline.

At the realization level, however, liquidation order alters the system-wide-collapse classification for some paired realizations. Random, larger-first, and smaller-first priorities produce classification flips in approximately 2.5–2.8% of these pairs, with flip rates reaching 4.8% at individual shock intensities. By contrast, the firms-first convention changes only one of the 8000 paired classifications. Thus, alternative liquidation orders can alter the system-wide-collapse classification of individual realizations while producing only small changes in the estimated system-wide collapse threshold.

#### 3.6.3. Feasibility of Baseline Configurations

The baseline experiment uses 101 shock intensities from 0.120 to 0.220, with 500 accepted realizations at each grid point. A new network and its associated balance sheets are generated for each realization. The generator checks the nonnegativity of residual bank deposits, firm fixed assets, and firm equity, and configurations that fail these checks are rejected. Of the 50,520 candidate systems generated for the baseline experiment, 20 are rejected, leaving 50,500 accepted realizations and a rejection rate of approximately 0.040%. These checks confirm that the accepted baseline configurations satisfy the feasibility conditions implemented in the model.

## 4. Conclusions

This study examines system-wide collapse in a three-layer financial network integrating interbank lending, bank–firm loans, and interfirm trade credit within a unified balance-sheet framework. In the baseline experiment, the bankruptcy fractions of both sectors exhibit pronounced bimodality, with the between-state component accounting for nearly all of the variance. The steep rise in mean bankruptcy fractions is therefore driven primarily by an increasing probability of system-wide collapse, rather than by a gradual upward shift in bankruptcy fractions across most realizations. Across five approximately degree-controlled system sizes, susceptibility peaks grow nearly linearly with sector size, while the shock-intensity interval over which the system-wide collapse probability rises from 10% to 90% generally narrows. Together, these results provide consistent finite-size evidence of phase-transition-like behavior in system-wide collapse.

The results also show that no single measure fully characterizes the system’s response to shocks. The system-wide collapse probability measures collapse likelihood at a given shock intensity, while the system-wide and sector-specific collapse thresholds characterize shock tolerance and differences between bank and firm responses. Layer-specific loss ratios provide complementary information on financial damage. In particular, losses may remain substantial without system-wide collapse and may continue to increase even after the system-wide collapse probability has reached one. Collapse likelihood, shock tolerance, and financial damage therefore describe distinct aspects of systemic risk in the model.

The comparative experiments further show how endogenous interfirm credit propagation and selected model parameters are associated with changes in shock tolerance. In the matched counterfactual experiments, the two-layer configurations without endogenous interfirm credit propagation have higher system-wide collapse thresholds than the three-layer model under both recovery-matching conventions, although the magnitude of the difference depends on how recovery resources are matched. The balance-sheet experiments show that higher bank capitalization and higher firm cash shares are associated with greater system-wide shock tolerance under the corresponding balance-sheet adjustments, while the sector-specific responses can differ substantially. In the interfirm-layer experiments, increasing $d_{f}$, which produces a denser interfirm network, is associated with a higher system-wide collapse threshold, whereas increasing the share of accounts payable in firms’ total assets is associated with a lower threshold. These results indicate that interfirm propagation, balance-sheet composition, link formation, and credit exposure are all associated with changes in the system-wide collapse threshold under the specified model configurations.

The main results are also robust to the methodological choices examined here. Reclassifying sectoral collapse using bankruptcy-fraction cutoffs of 0.90, 0.95, and 0.99 leaves the system-wide-collapse classifications unchanged in the baseline, finite-size, and matched-model experiments. Alternative liquidation orders produce only small changes in the estimated system-wide collapse threshold, although they alter the system-wide-collapse classification of some individual realizations. In addition, the accepted baseline configurations satisfy the feasibility conditions implemented in the balance-sheet generator.

These findings are conditional on the model’s specified network-generation, balance-sheet, shock, and contagion mechanisms. The generated networks are stylized configurations rather than empirically calibrated representations of actual financial systems, and the present analysis considers firm-originated shocks within a specified contagion process. It therefore does not establish whether the same patterns persist under alternative shock origins, targeting strategies, correlated shocks, or additional channels such as common asset exposures, fire sales, deposit runs, refinancing, and adaptive behavior. Further empirical calibration and extensions incorporating richer financial interactions and dynamic responses are needed to assess the broader applicability of the results.

## Figures and Tables

**Table 1:** Balance sheet of bank *i*.

Total Assets $A_{i}^{b}$	Total Liabilities $L_{i}^{b}$
Total loans to firms $L_{i}^{bf}$	Equity $E_{i}^{b}$
Interbank lending $L_{i}^{\mathrm{IBL}}$	Interbank borrowing $L_{i}^{\mathrm{IBB}}$
Cash $C_{i}^{b}$	Deposits $D_{i}^{b}$

**Table 2:** Balance sheet of firm *j*.

Total Assets $A_{j}^{f}$	Total Liabilities $L_{j}^{f}$
Accounts receivable $L_{j}^{AR}$	Equity $E_{j}^{f}$
Fixed assets $K_{j}^{f}$	Accounts payable $L_{j}^{AP}$
Cash $C_{j}^{f}$	Total bank loans $L_{j}^{fb}$

**Table 3:** Baseline parameter configuration of the multilayer financial network.

Parameter	Description	Value
$N_{b}$	Number of banks	200
$N_{f}$	Number of firms	3000
α	Pareto exponent	1.2
$A_{b}^{LB}$	Lower bound of bank total assets	100
$A_{b}^{UB}$	Upper bound of bank total assets	2000
$\lambda_{f}$	Average degree of firms in the bank–firm network	2
$\alpha_{1}$	Parameter governing link formation (common to interbank and interfirm networks)	0.25
$\alpha_{2}$	Parameter governing link formation (common to interbank and interfirm networks)	1
$d_{b}$	Parameter governing interbank link formation	0.5
$d_{f}$	Parameter governing interfirm link formation	0.5
$\omega_{b}$	Share of equity in total assets (banks)	0.1
$\theta_{bf}$	Share of loans to firms in total assets (banks)	0.5
$\theta_{bb}$	Share of interbank lending in total assets (banks)	0.2
$\mu_{fb}$	Share of bank loans in total assets (firms)	0.3
$\mu_{ff}$	Share of accounts payable in total assets (firms)	0.4
$\mu_{c}$	Share of cash in total assets (firms)	0.1
$\delta_{b}$	Bank liquidation recovery rate	0.7
$\delta_{f}$	Firm liquidation recovery rate	0.5

**Table 4:** Pre-shock characteristics of the baseline networks. Values are means across 150 independently generated networks.

Layer	Mean Out-Degree	Mean in-Degree	Density	Out–in Degree Correlation	Out-Degree–Asset Correlation	In-Degree–Asset Correlation
BF	33.815	2.254	0.01127	−0.0205	0.984	0.993
BB	9.512	9.512	0.04780	−0.0292	0.480	0.953
FF	94.092	94.092	0.03137	−0.0031	0.835	0.995

*Notes:* BF denotes the bank–firm layer, BB the interbank layer, and FF the interfirm layer. In the BF layer, out-degree refers to banks and in-degree to firms. The out–in degree correlation is the Pearson correlation between creditors’ out-degree and debtors’ in-degree across directed links within each layer. Degree–asset correlations are node-level Pearson correlations between the corresponding degree and pre-shock total assets.

**Table 5:** Baseline system-wide collapse thresholds and transition width, with 95% bootstrap confidence intervals. Confidence intervals are obtained from 1000 nonparametric bootstrap resamples.

Measure	Estimate	95% CI
$\varepsilon_{10}^{\mathrm{sys}}$	0.133571	[0.133222,0.134125]
$\varepsilon_{50}^{\mathrm{sys}}$	0.142629	[0.142358,0.142956]
$\varepsilon_{90}^{\mathrm{sys}}$	0.151600	[0.150611,0.152211]
$W_{10\text{–}90}$	0.018029	[0.016809,0.018767]

**Table 6:** Finite-size results under the degree-controlled protocol. The number of firms is set to $N_{f}=15N_{b}$. $\langle k_{bb}\rangle$ and $\langle k_{ff}\rangle$ denote the realized mean degrees of the interbank and interfirm layers, respectively, measured from pre-shock network realizations. Susceptibility peaks are the maxima observed on the simulated shock grid, and the system-wide collapse threshold is estimated by linear interpolation. The $N_{b}=200$ results are from an independent finite-size experiment and are not substituted for the baseline results in Section 3.1.

$N_{b}$	$d_{b}$	$d_{f}$	$\langle k_{\mathrm{bb}}\rangle$	$\langle k_{\mathrm{ff}}\rangle$	$\varepsilon_{50}^{\mathrm{sys}}$	$\chi_{b}^{\max}$	$\chi_{f}^{\max}$	$W_{10\text{–}90}$
150	0.6315	0.6303	9.354	93.739	0.144034	37.464	341.317	0.019096
200	0.5000	0.5000	9.437	94.681	0.142895	49.923	452.196	0.016640
250	0.4104	0.4108	9.378	93.563	0.142273	62.292	565.469	0.014673
300	0.3464	0.3551	9.336	95.042	0.142250	74.332	675.893	0.015590
400	0.2671	0.2758	9.348	93.570	0.141560	99.681	905.816	0.014455

**Table 7:** Matched three-layer and two-layer comparisons. The system-wide collapse threshold $\varepsilon_{50}^{\mathrm{sys}}$ and transition width $W_{10\text{–}90}$ are estimated from the observed system-wide collapse-probability curves using linear interpolation. Differences are defined as the two-layer value minus the three-layer value and are calculated before rounding.

Model	$\varepsilon_{50}^{\mathrm{sys}}$	$W_{10\text{–}90}$	$\Delta \varepsilon_{50}$	$\Delta W_{10\text{–}90}$
Three-layer baseline	0.142629	0.018029	–	–
Two-layer, rate-matched	0.187170	0.024214	+0.044541	+0.006186
Two-layer, amount-matched	0.150324	0.016327	+0.007694	−0.001701

**Table 8:** Sensitivity of the system-wide collapse threshold and classifications to liquidation order. Threshold differences are measured relative to the baseline order. The overall flip rate is the proportion of the 8000 paired realizations whose system-wide-collapse classifications differ between the alternative and baseline liquidation orders. The maximum flip rate is the largest such proportion at an individual shock intensity.

Liquidation Priority	$\varepsilon_{50}^{\mathrm{sys}}$	$\Delta \varepsilon_{50}$	Overall Flip Rate	Maximum Flip Rate
Baseline	0.142629	–	–	–
Random	0.142742	+0.000113	2.51%	4.4%
Larger first	0.142705	+0.000076	2.75%	4.8%
Smaller first	0.142694	+0.000065	2.61%	4.8%
Firms first	0.142629	0	0.0125%	0.2%

## Data Availability

No empirical datasets were used in this study. The numerical data generated and analyzed in the simulations, together with the MATLAB code used to produce the reported results, are provided in the Supplementary Materials. The code was developed and validated using MATLAB R2024b. Additional documentation describing the files and data structure is included with the supplementary code and data packages.

## Supplementary Materials

The following supporting information can be downloaded at https://www.mdpi.com/article/10.3390/e28101148/s1: Section S1: Pseudocode for the Default-Contagion and Liquidation Procedure (Algorithms S1 and S2); Section S2: Degree Distributions of the Baseline Networks (Figure S1); Section S3: Sensitivity to Liquidation Recovery Rates (Table S1 and Figure S2). The supplementary files also include the simulation code and numerical data used to generate the results reported in the article and Supplementary Materials. The code was developed and validated using MATLAB R2024b. Documentation, data dictionaries, and licensing information are included in the accompanying code and data packages.
