# Supplementary material for: Systemic Risk in a Multilayer Financial Network: A Criticality-Based Perspective
Source: Entropy (Basel). 2026 Oct 19;28(10):1148. doi: 10.3390/e28101148 (PMC13703311; doi:10.3390/e28101148)
Supplement: Supplementary file 1 [file entropy-28-01148-s001.zip › Supplementary_Materials.pdf]

Supplementary Materials

Systemic Risk in a Multilayer Financial Network:
A Criticality-Based Perspective

Ning Xi, Yamin Dou, and Yazheng Dang

S1. Pseudocode for the Default-Contagion and Liquidation Procedure

The following pseudocode summarizes one realization of the three-layer baseline model described in Section 2.5 of the main text. For compactness in the pseudocode, BB, BF, and FF denote the interbank, bank–firm, and interfirm layers, respectively. The generic term *claim* denotes a creditor's asset-side credit exposure; depending on the layer, it corresponds to interbank lending, a loan to a firm, or an account receivable. The corresponding debtor-side liabilities are interbank borrowing, bank loans, and accounts payable, respectively. All credit matrices are oriented from creditor to debtor.

A status of 0 denotes an active entity, 1 an entity that has been identified as bankrupt and awaits liquidation, and 2 a liquidated entity. Bankruptcy may be triggered either by negative equity or by insufficient cash when an outstanding claim is recalled. Balance-sheet variables and credit exposures are updated immediately after each transaction. Except for firms rendered insolvent directly by the initial shock, newly identified bankrupt entities are liquidated in the next contagion round. The pseudocode below follows the baseline liquidation order; alternative liquidation priorities are examined in Section 3.6.2 of the main text.

Algorithm S1. Initial shocks and iterative contagion (one realization).

Require: Accepted initial three-layer network, consistent bank and firm balance sheets, and shock-intensity target ε.

Ensure: Final bank and firm bankruptcy fractions and BB/BF/FF loss ratios.

Set all bankruptcy statuses to 0.
Set the BB, BF, and FF default-loss accumulators to zero.
Draw a uniform random permutation π of the firms.
Select the shortest prefix $J = (\pi_1, \dots, \pi_m)$ satisfying

$$\sum_{j \in J} A_j^f(0) \geq \varepsilon \sum_{j=1}^{N_f} A_j^f(0).$$

Record the realized shocked-asset share $\varepsilon_{\text{realized}}$.
for each firm j in the selected order J **do**
    Let $L \leftarrow C_j^f + K_j^f$, using the firm's current cash and fixed assets.
    Set $C_j^f \leftarrow 0$ and $K_j^f \leftarrow 0$.
    Reduce the firm's total assets and equity by L.
    if $E_j^f < 0$ **then**
        LiquidateFirm(j) immediately.
        Set $\text{status}_f(j) \leftarrow 2$.
    end if
end for
$Q_b \leftarrow \{i : \text{status}_b(i) = 1\}$.
$Q_f \leftarrow \{j : \text{status}_f(j) = 1\}$.
while $Q_b \neq \varnothing$ **or** $Q_f \neq \varnothing$ **do**
    Take snapshots of Q_b and Q_f.
    Process each snapshot in ascending entity-index order.
    for each bank i in the current Q_b **do**

LiquidateBank(i).
Set $\text{status}_b(i) \leftarrow 2$.
end for
for each firm j in the current Q_f **do**
LiquidateFirm(j).
Set $\text{status}_f(j) \leftarrow 2$.
end for
$Q_b \leftarrow \{i : \text{status}_b(i) = 1\}$.
$Q_f \leftarrow \{j : \text{status}_f(j) = 1\}$.
end while
Count entities with status 2 and compute ρ_b and ρ_f.
Normalize cumulative BB, BF, and FF default losses by their corresponding initial exposure totals.

Sequential claim-processing convention. When a bankrupt creditor recalls its outstanding claims, the claims are processed sequentially in ascending debtor-index order, as in the implementation. For a claim with face value x_{cd} from creditor c to debtor d, let C_d denote the debtor's current cash immediately before that claim is processed. The recovered amount is

$$p = \min\{x_{cd}, C_d\}.$$

The debtor pays p from current cash, and the corresponding liability and credit exposure are closed at the full face value x_{cd}. If $p < x_{cd}$, the debtor is identified as bankrupt because of the liquidity shortfall and is assigned bankruptcy status 1 unless it has already been liquidated. Because debtor balance sheets are updated immediately, later claims are evaluated using the remaining current cash rather than a beginning-of-round cash balance. Once a claim has been processed and closed, its unrecovered portion is not carried forward for a second recovery attempt. The bankrupt creditor's balance sheet is not further updated within the claim-collection routine; instead, the recovered amount p is accumulated for liquidation, while the unrecovered amount is recorded as default loss by the calling liquidation procedure.

Algorithm S2. Liquidation and repayment procedures.

procedure LiquidateFirm(j)
$A_{ff} \leftarrow$ current face value of firm j's outstanding accounts receivable.
$R_{ff} \leftarrow$ Collect(j, FF claims).
Add $A_{ff} - R_{ff}$ to the FF default-loss accumulator.
$F \leftarrow \delta_f(R_{ff} + C_j^f + K_j^f)$.
$B \leftarrow$ current bank-loan liabilities of firm j.
$T \leftarrow$ current accounts payable of firm j.
if $F \leq B$ **then**
$F_{bf} \leftarrow F$; $F_{ff} \leftarrow 0$.
else if $F < B + T$ **then**
$F_{bf} \leftarrow B$; $F_{ff} \leftarrow F - B$.
else
$F_{bf} \leftarrow B$; $F_{ff} \leftarrow T$.
end if
Add $B - F_{bf}$ to the BF default-loss accumulator.
Add $T - F_{ff}$ to the FF default-loss accumulator.
RepayClass(BF creditors of j, F_{bf}).
RepayClass(FF creditors of j, F_{ff}).
end procedure
procedure LiquidateBank(i)
$A_{bb} \leftarrow$ current face value of bank i's outstanding interbank lending.
$R_{bb} \leftarrow$ Collect(i, BB claims).

Add $A_{bb} - R_{bb}$ to the BB default-loss accumulator.
$A_{bf} \leftarrow$ current face value of bank i's outstanding loans to firms.
$R_{bf} \leftarrow$ COLLECT(i, BF claims).
Add $A_{bf} - R_{bf}$ to the BF default-loss accumulator.
$F \leftarrow \delta_b(R_{bb} + R_{bf} + C_i^b)$.
$D \leftarrow$ current deposit liabilities of bank i.
$B \leftarrow$ current interbank borrowing of bank i.
if $F \leq D$ **then**
$F_{bb} \leftarrow 0$.
else if $F < D + B$ **then**
$F_{bb} \leftarrow F - D$.
else
$F_{bb} \leftarrow B$.
end if
Add $B - F_{bb}$ to the BB default-loss accumulator.
REPAYCLASS(BB creditors of i, F_{bb}).
end procedure
procedure REPAYCLASS($\mathcal{C}, F_{\mathcal{C}}$)
Let x_c denote creditor c's outstanding claim in creditor class $\mathcal{C}$.
Set $X \leftarrow \sum_{c \in \mathcal{C}} x_c$.
if $X > 0$ **then**
for each creditor $c \in \mathcal{C}$ **do**
$p_c \leftarrow F_{\mathcal{C}} x_c / X$.
if c is a bank creditor in the BF layer **then**
Increase bank cash by p_c.
Remove the full claim x_c from its loans-to-firms asset.
Reduce its total assets and equity by $x_c - p_c$.
else if c is a bank creditor in the BB layer **then**
Increase bank cash by p_c.
Remove the full claim x_c from its interbank-lending asset.
Reduce its total assets and equity by $x_c - p_c$.
else
Increase firm cash by p_c.
Remove the full claim x_c from its accounts-receivable asset.
Reduce its total assets and equity by $x_c - p_c$.
end if
Close the corresponding claim–liability relationship.
if the creditor's updated equity is negative **then**
Set its bankruptcy status to 1 unless it has already been liquidated.
end if
end for
end if
end procedure
procedure COLLECT($c, \mathcal{D}$)
$R \leftarrow 0$.
Enumerate the positive claims x_{cd} in $\mathcal{D}$ by ascending debtor index d.
for each such claim x_{cd} **do**
Read debtor d's current cash C_d.
Set $p \leftarrow \min\{x_{cd}, C_d\}$.
Reduce the debtor's current cash by p.
Remove the corresponding liability at its full face value x_{cd}.
Close the processed claim–liability relationship.

if $p < x_{cd}$ **then**

Set debtor d's bankruptcy status to 1 unless it has already been liquidated.

end if

$R \leftarrow R + p$.

end for

return R.

end procedure

Implementation notes. The baseline three-layer model uses bank-first liquidation in each contagion round after the initial shocks, with entities processed in ascending index order within each sector. Within a bank liquidation, interbank lending is recovered before loans to firms. A bankrupt bank's liquidation fund is

$$F_i^b = \delta_b \left(R_i^{bb} + R_i^{bf} + C_i^b \right),$$

where R_i^{bb} and R_i^{bf} denote the amounts recovered from interbank lending and loans to firms, respectively. Deposits have priority over interbank borrowing.

For a bankrupt firm, accounts receivable are collected before the liquidation fund is formed:

$$F_j^f = \delta_f \left(R_j^{ff} + C_j^f + K_j^f \right),$$

where R_j^{ff} denotes the amount recovered from accounts receivable. Bank loans have priority over accounts payable. Within each creditor class, available repayment is distributed in proportion to outstanding face-value claims.

The claim-collection routines use only the debtor's current cash and do not recursively liquidate the debtor's claims or fixed assets to satisfy a recalled claim. If current cash is insufficient to satisfy a recalled claim in full, the debtor is identified as bankrupt because of the liquidity shortfall and enters a subsequent liquidation round. Balance sheets are updated after each processed claim, so the processing order can affect individual realizations. A processed claim–liability relationship is closed immediately and is not reconsidered in a later recovery attempt. The contagion procedure terminates when no entity remains with bankruptcy status 1.

S2. Degree Distributions of the Baseline Networks

Figure S1 presents the complementary cumulative degree distributions of the three network layers, based on 150 independently generated pre-shock networks. All links are directed from creditors to debtors. In the bank–firm layer, out-degree refers to banks and in-degree to firms; in the interbank and interfirm layers, both out-degree and in-degree are reported. These distributions provide descriptive diagnostics of the network structures generated under the baseline configuration and are not presented as evidence of a power-law form.

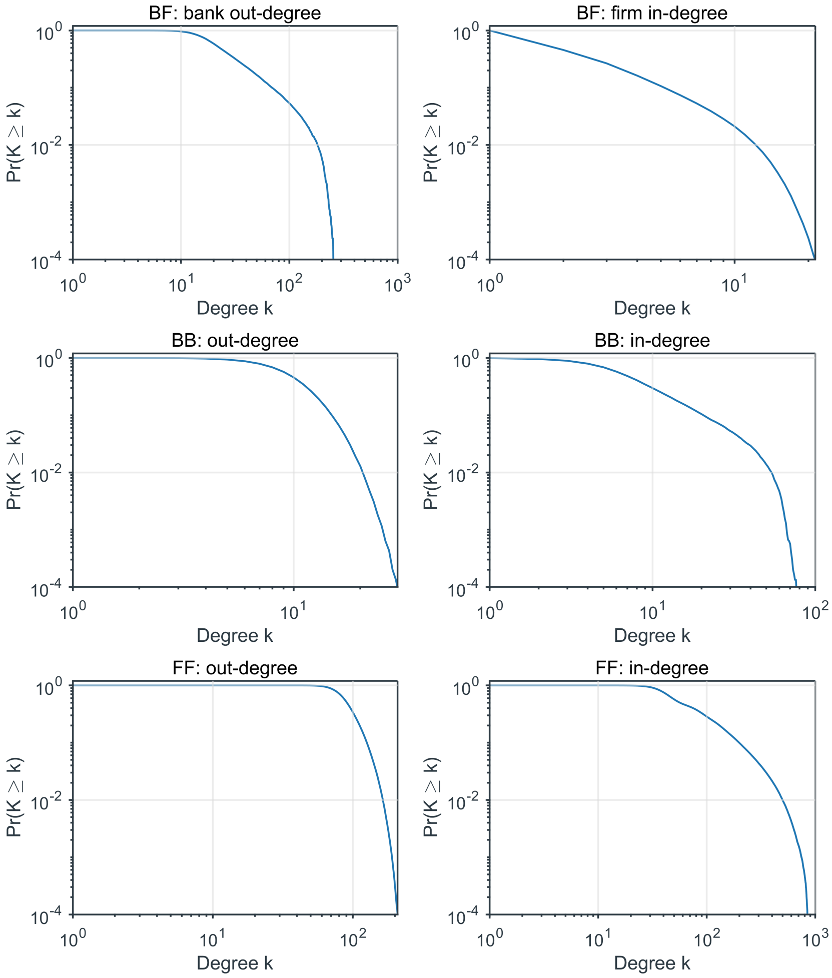

Figure S1: Complementary cumulative degree distributions of the three network layers. The bank–firm (BF) layer shows bank out-degree and firm in-degree; the interbank (BB) and interfirm (FF) layers each show out-degree and in-degree. Curves are based on 150 independently generated pre-shock networks. The distributions are reported descriptively and are not presented as evidence of a power-law form.

S3. Sensitivity to Liquidation Recovery Rates

We examine the sensitivity of the system-wide collapse probability and the system-wide collapse threshold to the two liquidation recovery rates defined in the main model: the bank liquidation recovery rate δ_b and the firm liquidation recovery rate δ_f. Their baseline values are $\delta_b = 0.7$ and $\delta_f = 0.5$. We vary one parameter at a time, considering $\delta_b \in \{0.5, 0.9\}$ while holding $\delta_f = 0.5$, and $\delta_f \in \{0.3, 0.7\}$ while holding $\delta_b = 0.7$. All other model parameters remain at their baseline values. These liquidation recovery rates govern the funds available during liquidation and should not be confused with the external recovery rate used in the matched two-layer counterfactuals in Section 3.3 of the main text.

At each simulated shock intensity, 500 accepted realizations are generated. Pilot simulations are used only to identify appropriate shock-intensity ranges, after which finer grids are used for the formal sensitivity experiments. The formal grids contain 56, 56, 57, and 92 shock-intensity points for the $\delta_b = 0.5$, $\delta_b = 0.9$, $\delta_f = 0.3$, and $\delta_f = 0.7$ configurations, respectively. For the $\delta_f = 0.7$ configuration, an additional 35 lower-shock-intensity points are simulated to extend the firm-sector analysis. These additional points lie below the range relevant for estimating the 0.5 crossing of the system-wide collapse probability curve and therefore do not change the interpolated estimate of $\varepsilon_{50}^{\text{sys}}$. The baseline curve is taken from the independent baseline experiment reported in Section 3.1 of the main text.

As in the main text, sectoral collapse is defined by a bankruptcy fraction of at least $q = 0.99$, and system-wide collapse occurs when both the banking and firm sectors satisfy this criterion. The system-wide collapse threshold $\varepsilon_{50}^{\text{sys}}$ is estimated by linear interpolation between the two adjacent simulated shock intensities that bracket the first upward crossing of $P_{\text{sys}} = 0.5$. We also report

$$W_{10\text{–}90} = \varepsilon_{90}^{\text{sys}} - \varepsilon_{10}^{\text{sys}},$$

the shock-intensity interval over which the system-wide collapse probability increases from 0.1 to 0.9. The 95% bootstrap confidence intervals reported below are calculated separately for each configuration and refer to the individual estimates of $\varepsilon_{50}^{\text{sys}}$.

Table S1: System-wide collapse thresholds $\varepsilon_{50}^{\text{sys}}$ and transition widths $W_{10\text{–}90}$ under alternative bank and firm liquidation recovery rates. The baseline experiment is independent of the four sensitivity configurations. Confidence intervals refer to individual estimates of $\varepsilon_{50}^{\text{sys}}$.

Configuration	δ_b	δ_f	$\varepsilon_{50}^{\text{sys}}$	95% CI	$W_{10\text{–}90}$
Baseline	0.7	0.5	0.142629	[0.142358, 0.142956]	0.018029
Lower δ_b	0.5	0.5	0.142724	[0.141973, 0.143417]	0.015569
Higher δ_b	0.9	0.5	0.142724	[0.142000, 0.143457]	0.015569
Lower δ_f	0.7	0.3	0.120125	[0.119722, 0.120517]	0.017919
Higher δ_f	0.7	0.7	0.324476	[0.323851, 0.325381]	0.019918

Table S1 and Figure S2 show that changing the bank liquidation recovery rate over the tested range has little effect on system-wide collapse probability or the system-wide collapse threshold. With $\delta_f = 0.5$, both $\delta_b = 0.5$ and $\delta_b = 0.9$ give $\varepsilon_{50}^{\text{sys}} = 0.142724$, close to the baseline estimate of 0.142629, and the corresponding system-wide collapse probability curves nearly overlap. This result is specific to collapse likelihood and shock tolerance as quantified here by the system-wide collapse probability and system-wide collapse threshold; it does not imply that δ_b has no effect on financial damage as measured by layer-specific loss ratios.

The system-wide collapse threshold is substantially more sensitive to the firm liquidation recovery rate over the tested range. Relative to the baseline value $\delta_f = 0.5$, lowering δ_f to 0.3 reduces $\varepsilon_{50}^{\text{sys}}$ from 0.142629 to 0.120125, whereas raising δ_f to 0.7 increases it to 0.324476. Despite these large changes in the system-wide collapse threshold, the corresponding values of $W_{10\text{–}90}$ remain of similar magnitude: 0.017919 for $\delta_f = 0.3$ and 0.019918 for $\delta_f = 0.7$, compared with 0.018029 under the baseline configuration.

At $\delta_f = 0.7$, $\varepsilon_{50}^{f} = 0.233821$ and $\varepsilon_{50}^{b} = 0.324476$; the banking-sector collapse threshold therefore determines the system-wide collapse threshold $\varepsilon_{50}^{\mathrm{sys}}$. This sectoral separation shows that the effect of a higher firm liquidation recovery rate differs across the two sectors. All collapse thresholds reported here are operational measures based on the prespecified bankruptcy-fraction cutoff $q = 0.99$. Because δ_b and δ_f are varied separately, these experiments constitute a one-factor sensitivity analysis and do not identify interactions between the two liquidation recovery rates.

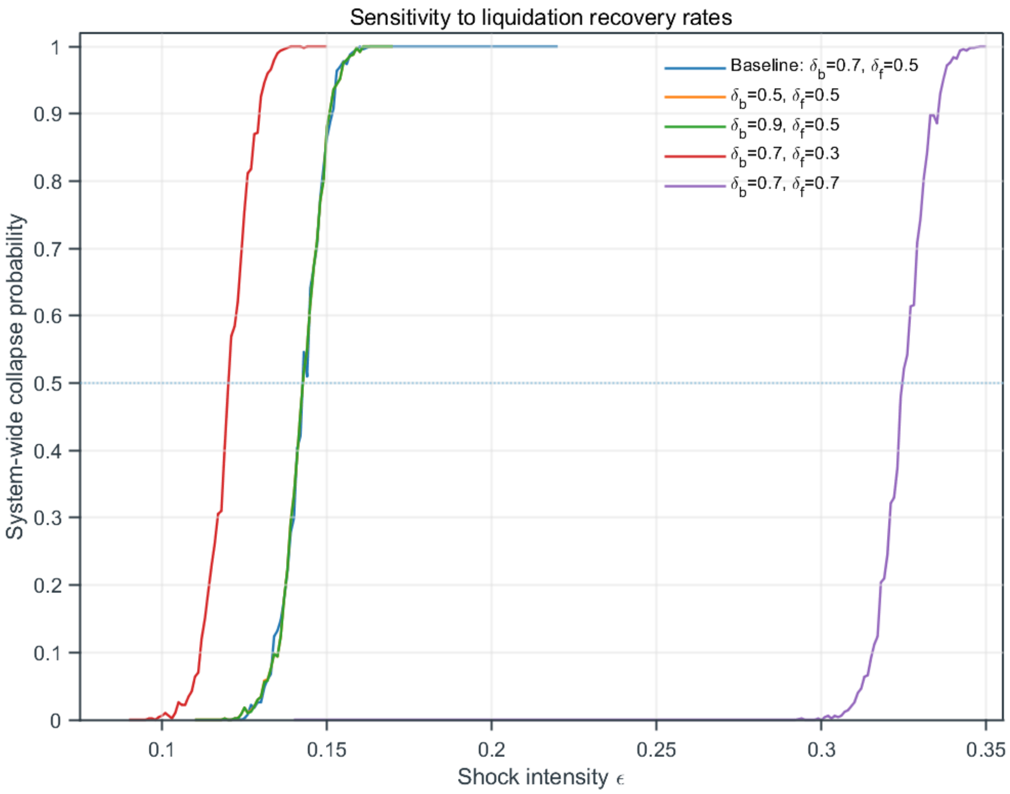

Figure S2: System-wide collapse probability as a function of shock intensity under the baseline liquidation recovery rates and four one-factor changes in δ_b or δ_f. Each plotted probability is estimated from 500 accepted realizations at the corresponding shock intensity. The baseline curve comes from the independent baseline experiment. The curves for $\delta_b = 0.5$ and $\delta_b = 0.9$ nearly overlap, whereas changes in δ_f produce substantial shifts in the system-wide collapse probability curve.
